# *Daucus carota DcPSY2* and *DcLCYB1* as Tools for Carotenoid Metabolic Engineering to Improve the Nutritional Value of Fruits

**DOI:** 10.3389/fpls.2021.677553

**Published:** 2021-08-26

**Authors:** Daniela Arias, Anita Arenas-M, Carlos Flores-Ortiz, Clio Peirano, Michael Handford, Claudia Stange

**Affiliations:** ^1^Centro de Biología Molecular Vegetal, Facultad de Ciencias, Universidad de Chile, Ñuñoa, Chile; ^2^Laboratorio de Nutrición y Genómica de Plantas, Instituto de Bioquímica y Microbiología, Facultad de Ciencias, Universidad Austral de Chile, Valdivia, Chile

**Keywords:** carotenoid, *Malus domestica*, *Solanum lycopersicum*, *DcPSY2*, *DcLCYB1*, *Daucus carota*, transgenic tomatoes, metabolic engineering

## Abstract

Carotenoids are pigments with important nutritional value in the human diet. As antioxidant molecules, they act as scavengers of free radicals enhancing immunity and preventing cancer and cardiovascular diseases. Moreover, α-carotene and β-carotene, the main carotenoids of carrots (*Daucus carota*) are precursors of vitamin A, whose deficiency in the diet can trigger night blindness and macular degeneration. With the aim of increasing the carotenoid content in fruit flesh, three key genes of the carotenoid pathway, phytoene synthase (*DcPSY2)* and lycopene cyclase (*DcLCYB1)* from carrots, and carotene desaturase (*XdCrtI)* from the yeast *Xanthophyllomyces dendrorhous*, were optimized for expression in apple and cloned under the *Solanum chilense* (tomatillo) polygalacturonase (PG) fruit specific promoter. A biotechnological platform was generated and functionally tested by subcellular localization, and single, double and triple combinations were both stably transformed in tomatoes (*Solanum lycopersicum* var. Microtom) and transiently transformed in Fuji apple fruit flesh (*Malus domestica*). We demonstrated the functionality of the *S. chilense* PG promoter by directing the expression of the transgenes specifically to fruits. Transgenic tomato fruits expressing *DcPSY2*, *DcLCYB1*, and *DcPSY2-XdCRTI*, produced 1.34, 2.0, and 1.99-fold more total carotenoids than wild-type fruits, respectively. Furthermore, transgenic tomatoes expressing *DcLCYB1*, *DcPSY2-XdCRTI*, and *DcPSY2-XdCRTI-DcLCYB1* exhibited an increment in β-carotene levels of 2.5, 3.0, and 2.57-fold in comparison with wild-type fruits, respectively. Additionally, Fuji apple flesh agroinfiltrated with *DcPSY2* and *DcLCYB1* constructs showed a significant increase of 2.75 and 3.11-fold in total carotenoids and 5.11 and 5.84-fold in β-carotene, respectively whereas the expression of *DcPSY2-XdCRTI* and *DcPSY2-XdCRTI-DcLCYB1* generated lower, but significant changes in the carotenoid profile of infiltrated apple flesh. The results in apple demonstrate that *DcPSY2* and *DcLCYB1* are suitable biotechnological genes to increase the carotenoid content in fruits of species with reduced amounts of these pigments.

## Introduction

**FIGURE 1:**
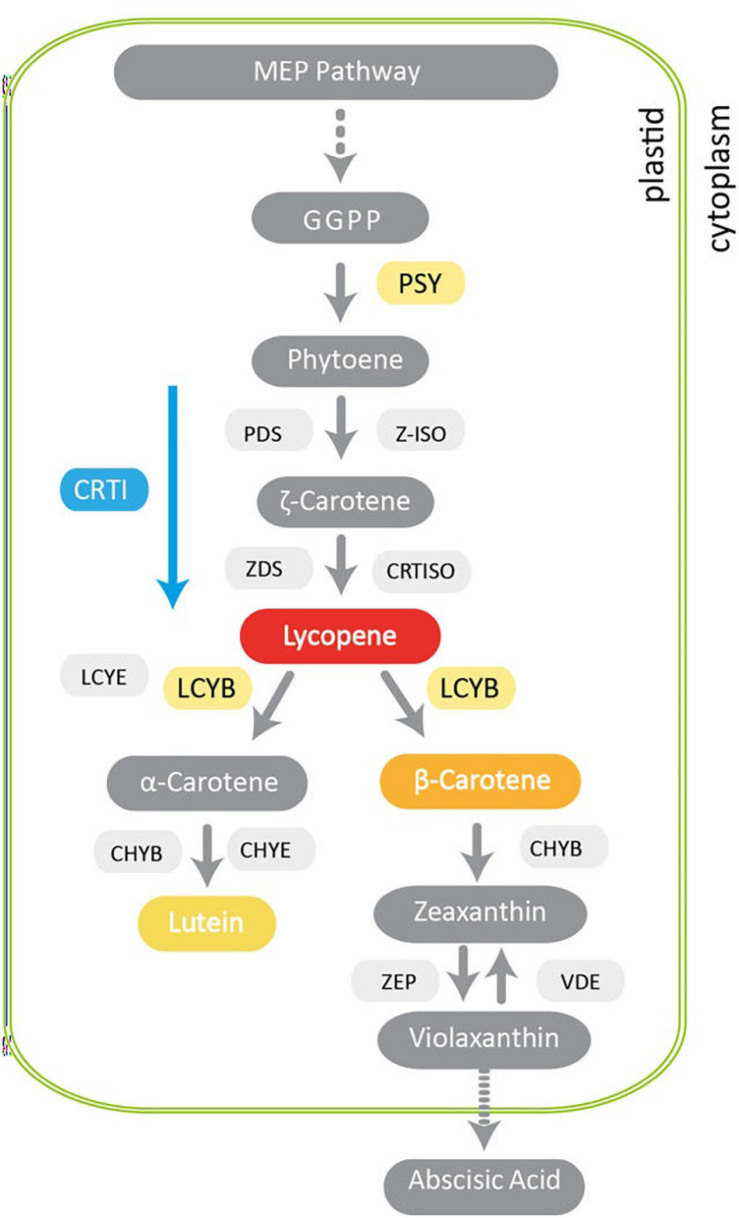
The main steps of carotenoid biosynthesis and related pathways in plants. The methylerythritol 4-phosphate (MEP) pathway produces isoprenoids in plastids leading to the production of geranylgeranyl pyrophosphate (GGPP). Phytoene synthase (PSY) is the first enzyme in the carotenogenic pathway and produces phytoene. In plants, phytoene is then desaturated and isomerized by phytoene desaturase (PDS), ζ–carotene desaturase (ZDS), ζ–carotene isomerase (Z-ISO) and prolycopene isomerase (CRTISO) forming lycopene. In bacteria, these reactions are carried out by a single multifunctional enzyme, phytoene desaturase (CRTI). β-carotene is produced if lycopene is cyclized only by lycopene β-cyclase (LCYB) whilst α-carotene is produced if both LCYB and lycopene ε-cyclase (LCYE) are involved. β-carotene hydroxylase (CHYB) and ε-carotene hydroxylase (CHYE) produce lutein. CHYB produces zeaxanthin, which can be converted into violaxanthin by zeaxanthin epoxidase (ZEP). The reverse step can be carried out by violaxanthin de-epoxidase (VDE). Violaxanthin is the precursor of abscisic acid. Dotted arrows indicate multiple enzymatic reactions. Solid gray arrows represent different steps of the carotenogenic pathway. The blue arrow indicates consecutive desaturations carried out by bacterial CRTI.

Carotenoids are natural lipophilic pigments produced by plant plastids that play important roles in light harvesting during photosynthesis and in protecting the photosynthetic apparatus against excessive light radiation. These secondary metabolites also normally accumulate in fruits, flowers and seeds, providing them yellow, orange, and red colors to facilitate pollination and seed dispersal (Rosas-Saavedra and Stange, 2016). Carotenoids are substrates for the biosynthesis of hormones that are crucial for plant physiology such as abscisic acid (ABA) and strigolactones, involved in the stress and developmental signaling responses (Walter and Strack, 2011; Beltran and Stange, 2016). Several plastidial enzymes have been identified that are essential for carotenoid synthesis. Phytoene synthase (PSY) catalyzes the first step and is the key regulatory point controlling flux through the pathway (Figure 1). The carotenoid pathway has been thoroughly described (Alcaíno et al., 2016). Briefly, the condensation of three molecules of isopentyl pyrophosphate (IPP) with one molecule of di-methylallyl pyrophosphate (DMAPP) generates the geranylgeranyl diphosphate (GGPP) precursor. The condensation of two molecules of GGPP, carried out by PSY, yields phytoene (C40). This is the first committed step in carotenoid synthesis, which is also highly regulated. In plants, colorless phytoene undergoes several steps of desaturation and isomerization by phytoene desaturase (PDS), ζ–carotene desaturase (ZDS), carotenoid isomerase (CRTISO) and ζ–carotene isomerase (ζ-ISO) (Isaacson et al., 2002, 2004; Lu and Li, 2008). To produce lycopene in bacteria and yeast, the four enzymatic steps from phytoene to lycopene mentioned above are carried out by a single enzyme (CRTI) (Alcaíno et al., 2016). Subsequently, lycopene is cyclized by lycopene β-cyclase (LCYB) producing β-carotene, whereas for α-carotene production, both LCYB and lycopene ε-cyclase (LCYE) are required. Then, the hydroxylation of α-carotene produces lutein, a yellowish pigment that is abundant in leaves (Hornero-Méndez and Britton, 2002; Bang et al., 2007; Misawa, 2011; Figure 1). Two subsequent hydroxylations of β-carotene, catalyzed by carotenoid β-hydroxylase (CHYB) produce zeaxanthin, which can be epoxidized by zeaxanthin epoxidase (ZEP) to form violaxanthin, which can be used by violaxanthin de-epoxidase (VDE) to regenerate zeaxanthin (Cazzonelli and Pogson, 2010; Cazzonelli, 2011; Moise et al., 2014). Finally, violaxanthin is the precursor for ABA, which is produced in the cytoplasm (Figure 1). In mammals, carotenoids possess antioxidant activity by quenching reactive oxygen species (ROS) (Stahl and Sies, 2003) thus decreasing the risk of developing certain diseases caused by oxidative stress (Malone, 1991; Byers and Perry, 1992; Voorrips et al., 2000; Holick et al., 2002) including cancer, atheromas in vascular diseases, aging and macular degeneration (Marnett, 1987; Mordi, 1993; Bartley and Scolnik, 1995; Rao and Rao, 2007). Carotenoids are also precursors of vitamin A. Specifically, the most relevant provitamin A carotenoids, given their high antioxidant activity and wide distribution in food, are α- and β-carotene, some xanthophylls such as β-cryptoxanthin and some *apo*-carotenoids (Mínguez-Mosquera and Hornero-Méndez, 1997). Of these, β-carotene presents the highest provitamin A activity since each molecule produces two retinal molecules that are then reduced to vitamin A (retinol). Vitamin A is converted into the visual pigment rhodopsin (retinal), in the retina of the eye, and acts as a co-regulator of gene expression (retinoic acid) (Zhu et al., 2013). Vitamin A is also required for cell growth and for healthy immunity, among other physiological processes (Olson, 1996). Therefore, increasing the carotenoid content in crop plants to improve nutritional value and benefits for human health has been a major goal of many research programs worldwide. Over the last four decades, significant progress has been made in the manipulation of carotenoid content and composition in a large number of crops by either genetic engineering or conventional breeding (Fraser et al., 1999, 2009; Giuliano et al., 2008; Rosati et al., 2009; Farré et al., 2010, 2011; Ye and Bhatia, 2012; Giuliano, 2014). Conventional and assisted breeding have successfully enhanced β-carotene levels in maize, sweet potato and cassava, benefiting human health in several countries (Ceballos et al., 2013; Pixley et al., 2013). Genetic engineering also allows nutritional traits to be targeted to specific organs (e.g., cereal seeds) and multiple traits can be combined in the same plants without complex breeding programs (Naqvi et al., 2009, 2010). Other examples of efficient enhancement of carotenoids through metabolic engineering have been obtained in agronomical relevant crops (Alós et al., 2016), such as rice (Paine et al., 2005), tomato (D’Ambrosio et al., 2004), potato (Diretto et al., 2006), carrot (Jayaraj et al., 2008), canola (Shewmaker et al., 1999; Ravanello et al., 2003), cassava (Failla et al., 2012), sorghum (Lipkie et al., 2013), orange (Pons et al., 2014), and apple (Arcos et al., 2020).

Apple (*Malus domestica*) is one of the most widely produced fruits in the world and is consumed not only as a fresh fruit but also in processed forms, such as juice and jam^1^ (Harker et al., 2003). As well as improving nutritional value, fruit color is one of the major aims of apple breeding because it provides novel appearances and influences consumer preferences (Yuan et al., 2014). The accumulation of anthocyanins and carotenoids in the skin and pulp are responsible for color in mature fruits (Telias et al., 2011; Cerda et al., 2020). However, commercial varieties such as “Royal Gala” and “Fuji” present low levels of carotenoids in the flesh reaching about 2-6 μg/g fresh weight (FW; Ampomah-Dwamena et al., 2012; Cerda et al., 2020). Specifically, the flesh of Fuji fruits accumulates around 1.8 μg/g FW in total carotenoids (Arcos et al., 2020), 0.04–0.22 μg/g dry weight (DW) of lutein and 0.79–1.14 μg/g DW of β-carotene (Delgado-Pelayo et al., 2014). Low carotenoid levels can be ascribed to reduced biosynthesis, deficient accumulation, and/or efficient degradation. Previous studies determined that the PSY enzyme in apple is codified by a family of genes with different levels of expression in fruit flesh (Ampomah-Dwamena et al., 2012; Cerda et al., 2020) and that the most expressed *PSY* genes are functional (Ampomah-Dwamena et al., 2015; Cerda et al., 2020) suggesting that the synthesis pathway is not the limiting step for carotenoid accumulation. In addition, the ectopic expression of the Arabidopsis 1-deoxy-D-xylulose 5-phosphate reductoisomerase (*AtDXR)* in Fuji apple, a gene participating in the synthesis of carotenoid precursors, produces a threefold rise in total and individual carotenoids in leaves (Arcos et al., 2020). This information led to the proposal that apple is a potential target to become a functional food with high pro-vitamin A and antioxidant components, when genes that codify for biosynthetic enzymes are expressed. Therefore, this work focused on the generation of a biotechnological platform for the rapid functional assessment of whether specific biosynthetic genes can be used to increase the content of provitamin A in fruits. This platform initially uses tomato fruits as a model, before final testing in apples.

Fruits and vegetables are the main sources of carotenoids, and among these, the orange carrot stands out. To generate the biotechnological platform, we chose *PSY2* and *LCYB1* from *Daucus carota* and *CrtI* from *Xanthophyllomyces dendrorhous.* Carrot accumulates high levels of α and β-carotene in its taproot, similar to *X. dendrorhous*, a yeast that is able to accumulate as much as 0.5% DW in astaxanthin, a ketocarotenoid produced from β-carotene (Niklitschek et al., 2008). Carrot presents two paralogs for *PSY* and *LCYB* genes (Just et al., 2007). *DcPSY2* and *DcLCYB1* are highly expressed in carrot leaves and tap roots during plant development (Fuentes et al., 2012; Simpson et al., 2016), suggesting functional roles for carotenoid synthesis. However, no functional characterization for *DcPSY2* has been published yet. Nevertheless, in the case of *DcLCYB1*, its overexpression results in 2–10 fold more carotenoids in transgenic tobacco (Moreno et al., 2016, 2020), and a threefold increase in carrots (Moreno et al., 2013). Indeed, DcLCYB1 also presents a plastidial localization (Moreno et al., 2013). Regarding *XdCrtI*, it codifies the enzyme that catalyzes the conversion of phytoene into lycopene in *X. dendrorhous* (Niklitschek et al., 2008). Although several studies have used *CrtI* from *Erwinia uredevora* to modify the synthesis of carotenoids in plants, as well as the use of other bacterial carotenogenic genes orthologous to *PSY* (*CrtB*) and *LCYB* (*CrtY*) (Römer et al., 2000; Ye et al., 2000; Fraser et al., 2002; Paine et al., 2005; Diretto et al., 2007), *XdCrtI* has yet to be used to increase carotenoids for this purpose. Here, we describe the design and development of carotenogenic gene expression vectors (pCP-CG) that utilize single or combinations of *DcPSY2* and *DcLCYB1* carrot carotenogenic genes and *XdCrtI* from yeast to increase the carotenoid level in apples. To guide transgenic gene expression, the selection of a suitable promoter is essential. In the case of fruits, one of the enzymes that participates in fruit ripening is polygalacturonase (PG), a hydrolase responsible for the degradation of polyuronides or pectins in the cell wall (DellaPenna et al., 1986). PG accumulates during fruit ripening, due to transcriptional activation of the *PG* gene (Sheehy et al., 1987; DellaPenna et al., 1989). The tomato PG promoter (pPG) was characterized by Montgomery et al. (1993), who showed that the first 806 pb upstream of the transcription start site are sufficient to confer fruit flesh (pulp) specific expression. Therefore, in this study, the carotenoid biosynthetic genes were cloned under the pPG from *Solanum chilense* (tomatillo). Once the biotechnological platform was built, the functionality of the vectors was evaluated through subcellular localization, by stable transformation of *Solanum lycopersicum* cv. Microtom and by transient expression in *M. domestica*. The results demonstrate that the pCP-CG vectors, specially pPSY2 and pLCYB1, increment the total carotenoid and β-carotene levels in tomatoes and/or apple fruits, proving their suitability in genetic engineering programs for the improvement of nutritional quality and color traits.

## Materials and Methods

### Vector Construction

pCP vector construction was based on a backbone from the pB7FWG2-AtDXR vector, which includes the p35S:DXR:GFP:T35S cassette (Perello et al., 2016, kindly provided by Dr. Manuel Rodriguez-Concepción, CRAG-CSIC, Spain). This cassette was obtained after a double digestion with *Xba*I and *Sac*I. The backbone fragment of 7,854 bp contains the RB and LB of the T-DNA and the *BAR* gene for BASTA or glufosinate (herbicide) resistance in plants. A specifically designed Multicloning Site (MCS) of 141 bp including *Eco*RI, *Apa*I, *Pst*I, *Avr*II, *Eco*RV, *Bsp*EI, *Nco*I, and *Bgl*II restriction sites flanked by *Xba*I and *Sac*I was synthesized by Genescript (United States^2^). The MCS was cloned in the *Xba*I and *Sac*I sites of the backbone to yield a new and royalty-free binary vector, named pCP that confers resistance to BASTA herbicide (Supplementary Figure 1, 201403013, Inapi, Chile).

**FIGURE 2:**
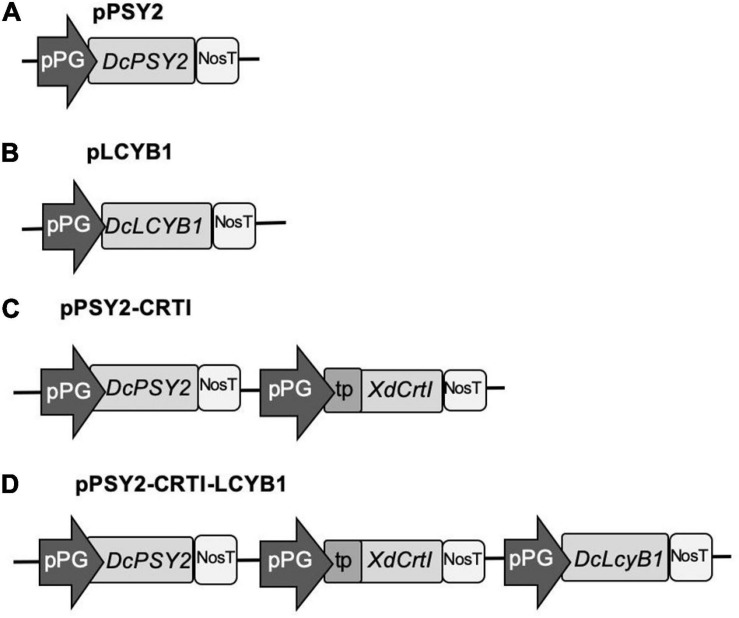
pCP-CG constructs used for the functional assessment of DcPSY2, DcLCYB1, and XdCRTI. The pCP backbone is derived from pB7FWG2.0, with the incorporation of a specifically designed Multiple Cloning Site (MCS; see Supplementary Figure 1). Different carotenogenic genes were cloned into the MCS, under the control of the tomato polygalacturonase promoter (pPG) and the Nos terminator (NosT). **(A)** pPSY2. The single gene and fruit-specific expression cassette PG:DcPSY2 was chemically synthesized and cloned into pCP. **(B)** pLCYB1. The single gene and fruit-specific expression cassette PG:DcLCYB2 was chemically synthesized and cloned into pCP. **(C)** pPSY2-CRTI. A double gene and fruit-specific expression cassette including the PG promoter driving the XdCRTI gene fused to a transit peptide (PG:tpXdCRTI) was chemically synthesized and cloned into pPSY2. **(D)** pPSY2-CRTI-LCYB1. The single gene and fruit-specific expression cassette PG:DcLCYB2 was cloned into pPSY2-CRTI.

DNA sequences of carrot *PSY2* (DQ192187) and *LCYB1* (DQ192190), and *CrtI* (gi.68250374) of *X. dendrorhous*, were optimized based on *M. domestica* codon usage by means of the OPTIMIZER software^3^. The *M. domestica* table obtained from^4^ was used as a reference set. Vector NTI software was used to verify the correct reading frame of the constructs as well as the amino acid homology with the native sequences encoded by *PSY2, CrtI*, and *LCYB1*. The FGENESH 2.6 gene prediction program was also employed, selecting the item ‘‘organism dicotyledonous plants^5^”. A plastidial signal peptide (tp) (171 bp) from the small subunit of pea Ribulose Bisphosphate Carboxylase Oxygenase (RUBISCO; EC 4.1.1.39 Seq ID X00806) was included at the 5′ NTR of *XdCrtI* to direct the encoded protein to these organelles (Figure 2). A 806 bp fragment of the tomato (*S. chilense*, tomatillo) PG promoter (pPG) was selected to direct the expression of each gene. A 277 bp fragment of the *Agrobacterium tumefaciens* nopaline synthase terminator (NosT) was included at the 3′ end of each coding sequence (Accession number V00087^6^). The sequences of interest, including pPG:DcPSY2:NosT, pPG:DcLCYB1:NosT, and pPG:tp:XdCrtI:NosT were synthesized and cloned in the pUC57 vector by GeneScript (United States). Afterward, pUC57:pPG:DcPSY2:NosT was digested with *Spe*I, pUC57:pPG:DcLCYB1:NosT with *Avr*II and pPG:tp:XdCrtI:NosT with *Xho*I, and each cassette was cloned into the MCS of pCP producing the single (pPSY2 and pLCYB1), double (pPSY2-CRTI) and triple vectors (pPSY2-CRTI-LCYB), collectively called pCP-CG vectors (Figure 2). Each vector was verified through sequencing (Macrogen Co.) and transformed into *Agrobacterium tumefaciens* (GV3101 strain). Positive clones were selected and used for stable transformation of *S. lycopersicum* var. Microtom and for transient transformation of *M. domestica* var. Fuji.

### Subcellular Localization of *DcPSY2* and *XdCrtI*

For the subcellular localization analysis, the full-length and optimized for *M. domestica DcPSY2* and *XdCrtI* sequences were amplified, including the PG promoter but excluding the stop codon from the previously sequenced pPSY2 and pPSY2-CRTI clones using primers listed in Supplementary Table 1. Amplicons were subcloned into pCR8/GW/TOPO (Invitrogen) and thereafter recombined into the binary vector pMDC107 (Curtis and Grossniklaus, 2003) in order to express the chimeric proteins DcPSY2:GFP and tp:XdCRTI:GFP, both under the control of pPG. As controls, the pPG:GFP (cloned in pCAMBIA 1302, Abcam) and the empty pMDC107 vector were included (Supplementary Figure 2). The constructs were transiently expressed by agroinfiltration in *Nicotiana tabacum* (tobacco) leaves and tomato fruits at breaker stage. *A. tumefaciens* suspensions containing the pPG:DcPSY2:GFP, pPG:tp:XdCRTI:GFP and both control constructs were infiltrated into 2 months old tobacco leaves and into the tomato fruit flesh following previously described protocols (Moreno et al., 2013; Arcos et al., 2020). Plants and fruits were maintained at 20–22°C, 30–50% humidity and while tobacco plants were kept under a 16 h white light photoperiod, tomato fruits were kept in the darkness for 4 days. Then, the infiltrated areas of the leaves and fruits were analyzed for GFP and chlorophyll fluorescence using a confocal LSM510 microscope. Images were processed with LSM5 Image Browser.

### Tomato Stable Transformation and *in vitro* Culture

*Solanum lycopersicum* var. Microtom was cultivated *in vitro* in solidified Murashige and Skoog (MS: 4.4 g/L MS salts, 20 g/L sucrose, and 0.7% agar-agar) medium and transformed according to a previous report (Pino et al., 2010). Briefly, cotyledons of *in vitro* 8–10 days post-germination seedlings were cut and dipped into an *A. tumefaciens* culture containing the respective genetic construct for 15–20 min, and then transferred into a co-culture medium (4.4 g/L MS salts, 30 g/L sucrose, 6 g/L agar-agar, and 100 μM acetosyringone) for 2 days in darkness at 25°C (Supplementary Figure 3). The explants were then transferred to the Induction medium (4.4 g/L MS salts, 100 mg/L Myo-Inositol, 30 g/L sucrose, 6 g/L agar, 5 μM BAP, 0.3 mg/L BASTA, and 400 mg/L Timentin) for callus induction and initial shoot development which started between 4 and 6 weeks post-transformation. The new shoots were transferred to Elongation medium (4.4 g/L MS salts, 100 mg/L Myo-Inositol, 15 g/L sucrose, 6 g/L agar, 0.5 mg/L BASTA, and 400 mg/L Timentin). After 2 months, 4–6 cm high plants developed several branches and a minimum of 4–5 leaves. Then, the shoots were transferred to Rooting medium (4.4 g/L MS salts, 100 mg/L Myo-Inositol, 15 g/L sucrose, 6 g/L agar, 0.5 mg/L BASTA, 400 mg/L Timentin, and 5 mg/L IBA) for 2 weeks. Three months-old plants developed a root system including primary and secondary roots, following which they were acclimated to greenhouse conditions (photoperiod of 16 h light/8 h darkness and illuminated with white fluorescent light; 150 μmol/m^2^ s at 22–25°C) in plastic pots (20 × 10 cm) containing a mix of soil and vermiculite (2:1), until they finally reached maturity and started producing fruits approximately 1 month later. Mature T1 plants were used for further analysis (Supplementary Figure 3).

### RNA Extraction and Quantitative Expression Analysis

Tomato RNA extraction was performed as described in Meisel et al. (2005). Briefly, mesocarps from five transgenic tomatoes (500 mg) were ground in liquid nitrogen and divided to perform three independent RNA extractions. The powder was then mixed with extraction buffer (2% CTAB, 2% PVP40, 25 mM EDTA, 2 M NaCl, 100 mM Tris–HCl, 0.05% spermidine, and 2% B-mercaptoethanol) at 65°C for 15 min. The samples were centrifuged and the phase containing the RNA was precipitated with 10 M LiCl overnight at 4°C. After centrifugation at 12,000*g* during 20 min, samples were resuspended in 20 μL of DEPC water. Genomic DNA traces were eliminated by a 40 min DNase I (Fermentas) treatment. For cDNA synthesis, 2 μg of DNA-free RNA were incubated with 1.5 μL of 10 μM oligo AP (Supplementary Table 1) and 8.3 μL of RT mixture (0.5 μL of RNase Inhibitor Ribolock, Fermentas), 1 μL of 10 mM dNTPs (Fermentas), 2.8 μL of 25 mM MgCl_2_, and 4 μL of 5× RT Improm II buffer (Promega) for 5 min at 70°C. Then, the samples were incubated for 5 min on ice. Subsequently, 1.2 μL of RT Improm II was added to continue with cDNA synthesis according to RT Improm II manufacturer’s instructions. For conventional expression analysis (Supplementary Figure 4), PCR reactions (28–35 cycles) were performed using Taq DNA polymerase (New England BioLabs) according to the manufacturer’s recommendations and an annealing temperature adjusted to each set of primers (Supplementary Table 1) and using *SlPSY* to show the cDNA quality. Quantitative expression analysis (qRT-PCR) was performed in a LightCycler system (MX3000P, Stratagene), using SYBR Green double strand DNA binding dye, as described in Fuentes et al., 2012). Specific primers for optimized *DcPSY2*, *DcLCYB1*, and *XdCRTI* were used (Supplementary Table 1), based on the optimized sequences (Supplementary Material). *Actin* was selected as reference gene. Each qRT-PCR reaction was performed with three biological and two technical replicates. In all cases, the reaction specificities were tested with melting gradient dissociation curves and non-template controls (NTC).

### Transient Transformation of Apple Fruits

Transient transformation of apples fruits was performed according to Arcos et al. (2020) with modifications. Fuji apple fruits at the mature green stage were infiltrated using the *Agrobacterium*-injection strategy containing the genetic construct of interest (pPSY2, pLCBY1, pPSY2-CRTI, or pPSY2-CRTI-LCYB1) and controls (WT). Each construct was infiltrated into a quarter of three fruits to have three biological replicas. Briefly, 30 mL of bacterial cultures in LB medium supplemented with Rifampicin (50 mg/L) and Spectinomycin (50 mg/L) were grown overnight at 28°C with agitation until an optical density (600 nm) of 0.2–0.3. The cultures were centrifuged, and the pellet resuspended in 30 mL of infiltration medium (10 mM MgSO_4_, 10 mM MES, 10 mM MgCl_2_). The culture was then infiltrated into the fruit flesh with a tuberculin syringe to a depth of 0.5 cm. For each apple, one half of the fruit was homogeneously agroinfiltrated. Agroinfiltrated fruits were maintained in an incubator with a photoperiod of 18 h light/6 h darkness, at 20–22°C and 30–50% of humidity. After 7 and 14 days post-infiltration, samples were taken for carotenoid quantification.

### Carotenoid Extraction and Quantification From Tomato and Apple Fruits

Mesocarps from five transgenic tomatoes (500 mg) of each line of approximately 40–45 days post-flowering were ground in liquid nitrogen and divided to carry out three independent carotenoid extractions. For transient analysis, three independent apple fruits were agroinfiltrated with each construct including both controls, and after 7 and 14 days, 300 mg of infiltrated flesh of each apple were ground in liquid nitrogen independently. In all cases, each sample was measured twice, by adding 4 mL of hexane:acetone:ethanol (2:1:1) to obtain an homogeneous mixture. This solution was transferred to a tube, shaken for 2 min, incubated on ice and darkness for a further 2 min, and then centrifuged at 10.000 rpm for 10 min at 4°C. Finally, the carotenoids were recovered from the upper phase and dried using gaseous nitrogen. Once dried, the samples were stored at −80°C or resuspended in 2 mL of acetone for carotenoid quantification. All extractions were carried out in on ice and in dim light conditions. Total carotenoids were quantified was carried out using a Shimadzu spectrophotometer at 474, 645/662 (chlorophyll *a* and *b* contribution) and at 520/750 nm (for turbidity). Quantification of individual carotenoids was undertaken in a Reverse Phase High Performance Liquid Chromatography (RP-HPLC) using a RP−18 Lichrocart 125−4 reverse phase column (Merck^®^). Pigment composition was determined according to Lichtenthaler and Buschmann (2001). The mobile phase was acetonitrile:methanol:isopropyl alcohol (85:10:5) with a 1.5 mL/min flow rate at room temperature in isocratic conditions. Carotenoid and chlorophyll concentrations were calculated according to the total pigments obtained at 474 nm by spectrophotometry, by retention times, absorption spectra and purity of the peaks, as described in Fuentes et al. (2012) and corroborated with the Carotenoids Handbook (Britton, 1995; Britton et al., 2004).

## Results

### Modifications of Carotenogenic Genes and Functional Analysis of pCP-CG Vectors by Subcellular Localization

To generate the biotechnological platform to boost carotenoid content in apples, we chose *PSY2* and *LCYB1* from *D. carota* and *CrtI* from *X. dendrorhous.* The codon usage of each gene was optimized to maximize expression in apples (Supplementary Material).

The *XdCrtI* was destined to plastids using the transit peptide (tp) of the RUBISCO small subunit (Supplementary Material). This transit peptide has been used to direct *Erwinia uredovora* CrtI to tobacco leaf plastids, as determined by immunolocalization with gold particles (Misawa et al., 1993). We also amplified a 806 bp fragment upstream of the transcription start site of the PG promoter from *S. chilense* (tomatillo) (Supplementary Material) in order to direct expression of the carotenogenic genes to fruits. Using the different modules – the three carotenogenic genes, the tp and the PG promoter – four pCP-CG vectors were generated with different gene combinations, as described in section “Materials and Methods” (Figure 2).

**FIGURE 3:**
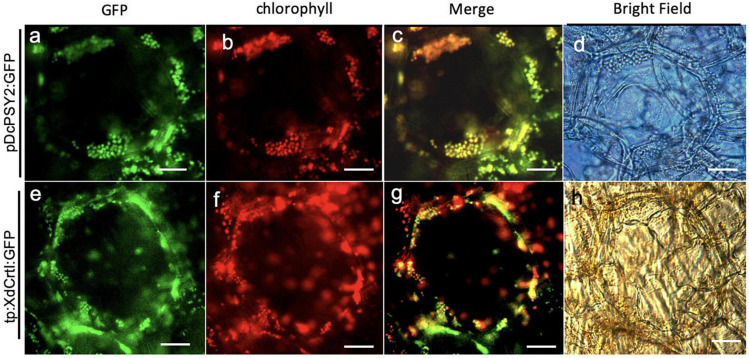
Subcellular localization of DcPSY2 and XdCRTI. Transient transformation of tomato fruits agroinfiltrated with DcPSY2:GFP or XdCrtI:GFP fused to a transit peptide (tp). The expression of both constructs was driven by the pPG promoter. Fluorescence was observed 4 days after transient transformation. **(a)** GFP fluorescence of DcPSY2:GFP. **(b)** Chlorophyll autofluorescence of **(a)**. **(c)** Merge of panels **(a,b)**, showing colocalization of chlorophyll and the GFP signal. **(d)** Bright field of **(a–c)**. **(e)** GFP fluorescence of tp:XdCRTI:GFP. **(f)** Chlorophyll autofluorescence of **(e)**. **(g)** Merge of panels **(e,f)**, showing colocalization of chlorophyll and GFP signal. **(h)** Bright field of **(e–g)**. The TRITC filter U-N31001 was used for visualizing GFP fluorescence, with an excitation wavelength between 465 and 495 nm. Emission was collected between 513 and 556 nm. Chlorophyll autofluorescence was assessed by using a U-MNG2 filter, with an excitation wavelength between 530 and 550 nm and emission collected at 590 nm. Scale bar = 50 μm.

Subsequently, we determined the correct plastid location of DcPSY2 and tp:XdCrtI; that of DcLCYB1:GFP has been reported previously (Moreno et al., 2013). Therefore, pPG:PSY2:GFP and pPG:tp:XdCrtI:GFP were transiently expressed in tomato fruits at breaker stage where both chloroplasts and chromoplasts are present in the flesh of the fruit. GFP fluorescence showed that DcPSY2:GFP had a speckled distribution (Figure 3a). This signal colocalized with the emission of chlorophyll (Figures 3b,c), strongly suggesting that DcPSY2:GFP has a chloroplastic localization. Similarly, GFP signal from tp:XdCrtI:GFP exhibited a distribution pattern of discrete foci (Figure 3e), which colocalized with the autofluorescence from chlorophyll (Figures 3f,g). Therefore, this result showed that the plastidial tp from the pea RUBISCO small subunit was functional in tomato. Considering that the GFP fluorescence distribution following infiltration with the control pPG:GFP construct was cytoplasmic in fruits, and much reduced in tobacco leaves (Supplementary Figure 2), we demonstrated the fruit specificity of the PG promoter from *S. chilense*. Overall, these results show that DcPSY2:GFP and tp:XdCrtI:GFP are chloroplastic.

### Stable Transformation of *S. lycopersicum* With the pCP-CG Vectors Produce Fruits With Higher Carotenoid Contents

The functional characterization of pPSY2, pLCYB1, pPSY2-CRTI, and pPSY2-CRTI-LCYB1 vectors was carried out by stable transformation of *S. lycopersicum* var. Microtom. The *in vitro* transformation and regeneration culture to obtain transgenic plants was adapted from the protocol described in Pino et al. (2010) and summarized in Suppplementary Figure 3. During plant development, no phenotypic differences were noticeable with respect to WT tomato plants, except in the case of those transformed with pPSY2-CRTI-LCYB1 that showed dramatic alterations at all stages during the regeneration process (Suppplementary Figure 3). These lines exhibited a slower induction of shoots and their vegetative development was also delayed in comparison with the other transgenic lines. Interestingly, these lines produced tomatoes when they were still *in vitro*, about 2 months after the regeneration process started. This was significantly earlier in comparison with the WT and other transformed lines, which only produced fruits once they were in soil in greenhouse conditions.

**FIGURE 4:**
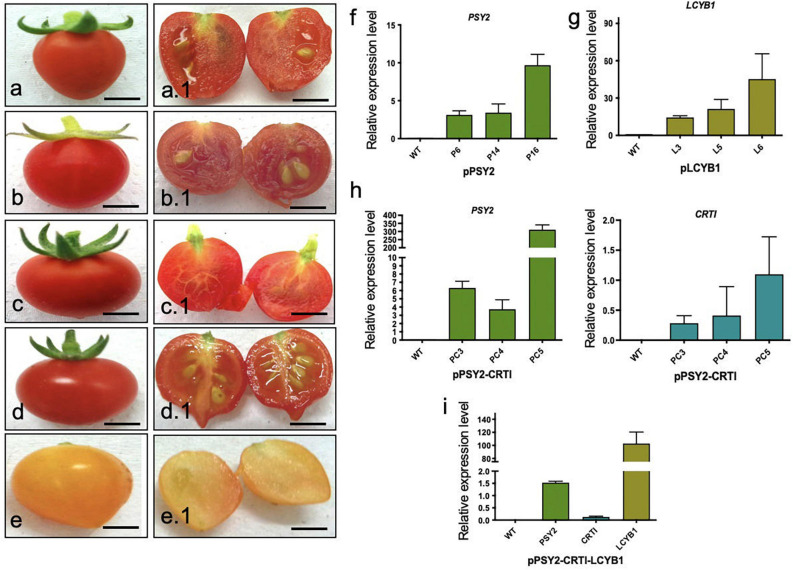
Fruit phenotypes and expression analysis in T1 transgenic fruits. **(a)** Representative WT tomato fruit. **(a.1)** Cross-section of **(a)**. **(b)** Representative tomato fruit from pPSY2 line. **(b.1)** Cross-section of **(b)**. **(c)** Representative tomato fruit from pLCYB1 line. **(c.1)** Cross-section of **(c)**. **(c)** Representative tomato fruit from pPSY2-CRTI line. **(d.1)** Cross-section of **(d)**. **(e)** Tomato fruit from pPSY2-CRTI-LCYB2. **(e.1)** cross-section of **(e)**. Scale bar = 1 cm. **(f)** q-RTPCR in fruits of pPSY2, **(g)** pLCYB1, **(h)** pPSY2-CRTI, and **(i)** pPSY2-CRTI-LCYB1 transgenic lines. The relative expression was normalized to that of the *Actin* gene of *S. lycopersicum*. The expression analysis was performed with three biological replicas (cDNAs) from five fruits, each measured twice.

**FIGURE 5:**
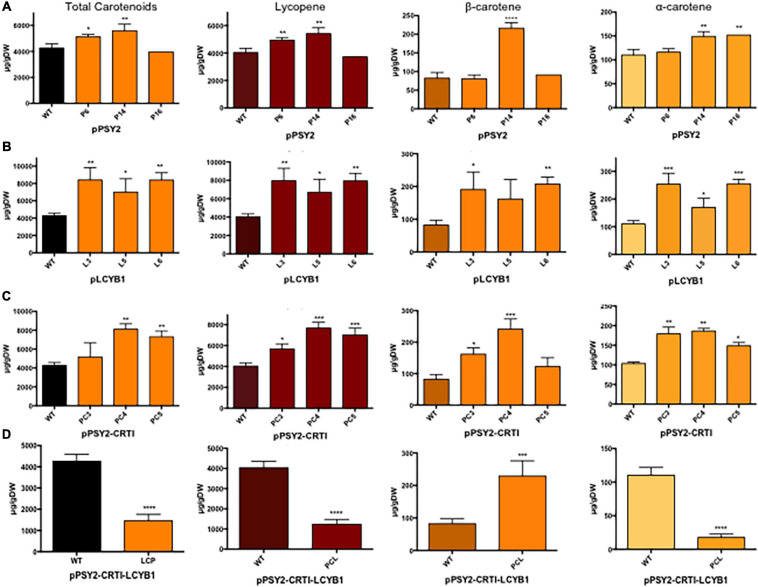
Total and individual carotenoid quantification in pCP-CG transgenic lines. **(A)** pPSY2, **(B)** pLCYB1, **(C)** pPSY2-CRTI, and **(D)** pPSY2-CRTI-LCYB1 transgenic lines. Total carotenoids were quantified in fruits by spectrophotometry at 474 nm and individual carotenoids by HPLC expressed in μg/gDW. Asterisks indicate significant differences respect to the control (WT) determined by one-way ANOVA and Dunnet post-test, *p < 0.05, **p < 0.01, ***p < 0.001. In **(D)** the results were analyzed with a two-way *t*-test, ****p < 0.0001.

Transformed tomato plants were subjected to molecular analysis to select transgenic lines once they were acclimated to the greenhouse conditions, except for pPSY2-CRTI-LCYB1. Twelve pPSY2 lines, seven pLCYB1 lines, and eight pPSY2-CRTI lines were transgenic, based on the respective transgene amplification (data not shown). At fructification, tomato fruits from untransformed WT plants, as well as from lines transformed with pPSY2, pLCYB1, and pPSY2-CRTI were observed at different developmental and ripening stages ranging from incipient green fruits to red, fully mature fruits; no significant differences between them in terms of size and ripening time were noted, and all were similar to previous reports (Fraser et al., 2002; Apel and Bock, 2009). However, in the case of the pPSY2-CRTI-LCYB1 construct, it was not possible to regenerate fully developed plants; instead stem-like structures were obtained with a reduced number of incipiently developed leaves. Nonetheless, these plants showed an accelerated flower development with fully functional flowers that yielded fruits during *in vitro* culture (Suppplementary Figure 3). Since the tissue was limited, molecular analysis of these fruits was challenging. Thus, transgene expression was confirmed when some of these fruits reached maturity. We determined that fruits of seven pPSY2 lines, five pLCYB1 lines and seven pPSY2-CRTI lines expressed the transgene (Supplementary Figure 4), and three of each were selected for qRTPCR and phenotypic analysis (Figure 4) and carotenoid quantification (Figure 5). As explained above, it was not possible to regenerate fully developed lines transformed with pPSY2-CRTI-LCYB1. Therefore, the expression analysis was performed with a group of tomatoes obtained from *in vitro* culture and not from individual transgenic lines (Figure 4 and Supplementary Figure 4).

For these evaluations, five mature fruits of each line were harvested and phenotypically analyzed. Fully ripe WT tomato fruits (2.2 ± 0.3 cm diameter) have a homogeneous red exocarp, are firm to the touch and have a central pedicel surrounded by several sepals (Figure 4a). Transverse sections showed a red fleshy mesocarp and endocarp with several yellowish seeds embedded in a locular cavity (Figure 4a.1). Transgenic tomato fruits from pPSY2 (Figures 4b,b.1), pLCYB1 (Figures 4c,c.1), and pPSY2-CRTI (Figures 4d,d.1) were phenotypically similar to fruits from WT plants (2.4 ± 0.3 cm diameter). On the other hand, pPSY2-CRTI-LCYB1 transgenic fruits exhibited a pale orange color at the ripening stage (Figures 4e,e.1), unlike the fruits of the WT and other transgenic lines, which turned red. In addition to the color difference, these transgenic fruits did not develop seeds and were therefore sterile lines (Figure 4e.1). Quantitative expression analysis showed that pPSY2 lines (P6, P14, and P16) present between 2.8 and 9.8-fold of *PSY2* expression, pLCYB1 lines (L3, L5, and L6) present between 12 and 45.2-fold *LCYB1* expression (Figures 4f,e, respectively), whereas pPSY-CRTI lines (PC3, PC4, and PC5) present between 3.6 and 300-fold expression of *PSY2* and 0.2 and 1.2-fold *CRTI* expression (Figure 4h). In the case of the transgenic pPSY2-CRTI-LCYB1 fruits, *CRTI* expression was significantly lower than that of *PSY2* and *LCYB1* (Figure 4i).

Regarding the total content of carotenoids, and compared to WT plants, the pPSY2 lines present a 1.21–1.34-fold increase, pLCYB1 lines present a 1.7-2-fold increase, and pPSY2-CRTI lines showed a 1.21–1.99-fold increase whilst pPSY2-CRTI-LCYB1 lines had a 2.9-fold decrease (Figure 5). This suggests that the expression of *LCYB1* is sufficient to enhance the total carotenoid content in fruits and that the simultaneous expression of these three transgenes negatively modulates the carotenoid pathway, reducing the total carotenoid content in tomato fruits (Figure 5).

Analysis of the individual carotenoid profiles revealed that transgenic tomatoes from the pPSY2 lines had up to 1.25-fold more lycopene, and only one line presented a 2.5-fold increase in β-carotene and two had a 1.27-fold increment in α-carotene with respect to WT (Figure 5A). Transgenic fruits from the pLCYB1 lines had a 1.75–2.0-fold increase in lycopene, 2.12–2.5-fold increase in β-carotene, and 1.36-2.27 fold increment in α-carotene with respect to the WT (Figure 5B). Fruits of pPSY2:CRTI lines presented a 1.87-1.95-fold increase in lycopene, two to threefold increase in β-carotene and 1.4-1.75-fold increment in α-carotene with respect to WT (Figure 5C), whereas pPSY2-CRTI-LCYB1 transgenic tomatoes present 3.6-fold less lycopene, a 2.57-fold increment in β-carotene, and 7.3-fold less α-carotene compared to untransformed controls (Figure 5D). The orange coloration phenotype of these tomatoes could be due to the combination of a reduced amount of red lycopene together with an increment in orange β-carotene (Figure 4e). These results suggest that the expression of carrot carotenogenic genes is capable of modulating carotenoid synthesis in a fruit that naturally accumulates large amounts of these pigments. Moreover, the increase in carotenoid content does not directly correlate with the expression level, as exemplified by comparing pPSY2 and pPSY2-CRTI lines. Interestingly, pLCYB1 was the genetic construct that triggered the highest increase in carotenoid content, constituting the most promising tool for future experiments.

### Carotenoid Quantification in Apple Fruits After Transient Expression of pCP-CG Vectors

In order to test if the pCP-CG constructs were able to modify the content of carotenoids in apple, transient assays were carried out in Fuji fruits at breaker stage (Suppplementary Figure 5). At 7 and 14 days post-agroinfiltration, tissue samples were taken for carotenoid analysis. After 7 days of incubation, apples agroinfiltrated with pPSY2 and pLCYB1 showed an increase of 1.65 to 1.50-fold in total carotenoid content (4.3 and 3.94 μg/gFW, respectively) in comparison to non-agroinfiltrated (WT) apples (2.61 μg/gFW) (Table 1). Carotenoid profile analysis revealed that for these constructs, the lutein levels remain stable with respect to the control (Table 1). On the contrary, the β-carotene content almost doubles (to 3.51 μg/gFW in pPSY2 and to 3.30 μg/gFW in pLCYB1), respect to the WT apple fruits (1.81 μg/gFW) (Table 1). Fruits agroinfiltrated with pPSY2-CRTI showed a significant increment in total carotenoids and β-carotene of 1.17- and 1.3-fold (3.05 and 2.35 μg/gFW, respectively) (Table 1). Similarly, apple fruits infiltrated with pPSY2-CRTI-LCYB1 exhibited an increment in their total carotenoid (3.07 μg/gFW) and β-carotene (2.23 μg/gFW) content in comparison to control WT fruits, but to a similar or lesser extent in comparison with fruits agroinfiltrated with other pCP-CG constructions (Table 1).

**TABLE 1:** Quantification of carotenoids in apple fruits agroinfiltrated with the pCP-CG vectors.

Days post-infiltration	Fuji Apple	Total carotenoid (μg/gFW)				Lutein (μg/gFW)				β-carotene (μg/gFW)			
				
		Mean	SD	FC	*t*-test	Mean	SD	FC	*t*-test	Mean	SD	FC	*t*-test
7	WT	2.62	0.11	**–**	–	0.81	0.14	**–**	–	1.81	0.03	–	–
	pPSY2	4.31	0.32	**1.65**	**	0.79	0.07	**–**	ns	3.51	0.26	**1.94**	***
	pLCYB1	3.94	0.31	**1.50**	**	0.64	0.11	**–**	ns	3.30	0.31	**1.82**	**
	pPSY2-CRTI	3.05	0.22	**1.17**	*	0.70	0.10	**–**	ns	2.35	0.23	**1.30**	*
	pPSY2-LCYB-CRTI	3.08	0.17	**1.18**	*	0.84	0.15	**–**	ns	2.23	0.18	**1.23**	*
14	WT	2.06	0.30	**–**	–	1.12	0.16	**–**	–	0.94	0.19	**–**	–
	pPSY2	5.67	1.60	**2.75**	*	0.88	0.27	**–**	ns	4.79	1.37	**5.11**	**
	pLCYB1	6.39	1.33	**3.11**	**	0.92	0.20	**–**	ns	5.47	1.16	**5.84**	**
	pPSY2-CRTI	4.67	0.77	**2.27**	**	1.27	0.42	**–**	ns	3.40	0.53	**3.63**	**
	pPSY2-LCYB-CRTI	4.27	0.63	**2.07**	**	0.83	0.06	**−0.74**	*	3.44	0.69	**3.67**	**

*Values are means of three biological replicas. “FW” represents Fresh Weight, “SD” Standard deviation and “FC” Fold of Change. Significant levels: ^∗^*P* < 0.05; ^∗^^∗^*P* < 0.01; ^∗^^∗^^∗^*P* < 0.001; ns: not significant; – not considered. The bold values represent the pigments fold of change (FC) between agroinfiltrated apples respect to non-agroinfiltrated apples (WT).*

Samples taken 14-days post-infiltration revealed that the total carotenoid content doubled in fruits agroinfiltrated with each one of the four pCP-CG constructs in comparison to WT (Table 1). Similarly, a significant increase in β-carotene levels was found in the infiltrated fruits with the pCP-CG series (4.78 μg/gFW for pPSY2, 5.47 μg/gFW for pLCYB1, 3.40 μg/gFW for pPSY2-CRTI, and 3.43 μg/gFW for pPSY2-CRTI-LCYB1) in comparison to control WT fruits (0.94 μg/gFW; Table 1). On the other hand, the apple fruits transiently transformed with the pCP-CG constructs did not present changes in the lutein content (Table 1), maintaining the same correlation observed in the 7-day incubation experiment. Altogether, these results suggest that transient expression of the pCP-CG constructs are functional in apple fruits, and suggest that double or triple constructs with genes arranged in tandem do not trigger a synergistic effect on the carotenoid content.

## Discussion

### Biotechnological Platform

Significant progress has been made in the generation of genetically modified crops with improved carotenoid content and nutritional properties (Alós et al., 2016). With the same goal, we designed and generated a biotechnological platform to evaluate key genes, with the aim of improving the carotenoid content, and thus the nutritional value of apples. The selection of the carotenogenic genes was based on our previous studies regarding carotenoid biosynthesis regulation in carrot, particularly in the taproot, which accumulates up to 1.5 mg/gDW in total carotenoids (Fuentes et al., 2012; Moreno et al., 2013, 2016; Rodriguez-Concepcion and Stange, 2013; Simpson et al., 2016, 2018). We have established that the increment in carotenoids correlates with a significant increase in the expression of almost all carotenogenic genes, especially *DcPSY2, DcZDS2, DcPDS, DcLCYB1, DcLCYE, DcZEP*, and *DcNCED1* (Simpson et al., 2016). The two *PSY* paralogs in carrot, *DcPSY1* and *DcPSY2*, present an induction of 3.2 and 316-fold during carrot tap root development, respectively, whereas *LCYB1* rises by eightfold and *LCYB2* by 100-fold at the mature stage of taproot development (Simpson et al., 2016).

Some of the carotenogenic genes from carrot have been previously characterized, demonstrating that they are functional *in vivo*. Both *DcZDS1* and *DcLCYB1* code for functional enzymes necessary for carrot development and carotenoid synthesis (Moreno et al., 2013; Flores-Ortiz et al., 2020). Total carotenoids rise threefold when *DcLCYB1* is overexpressed in carrot and a 2–10-fold increment when expressed in tobacco, with concomitant increases in chlorophyll content and photosynthetic activity which together have a positive impact on plant fitness (Moreno et al., 2013, 2016, 2020). The ability of LCYB1 to confer these qualities by itself when expressed in plants was the reason for selecting this gene as a candidate to be assessed in the biotechnological platform developed in this work. On the other hand, *DcPSY2* was selected as previous data suggested that it could play a relevant role during the stress response; we determined that its expression is induced by saline stress and ABA in carrot, and showed that AREB transcription factors bind to the *DcPSY2* promoter (Simpson et al., 2018). Here we demonstrated that DcPSY2:GFP has the expected plastidial subcellular localization (Figure 3), and is therefore a possible candidate for boosting carotenoid content in both transgenic pPSY2 tomatoes (Figure 5A) and transiently infiltrated apple fruit flesh (Table 1). In the case of *XdCrtI*, its functionality was demonstrated by site directed mutagenesis using the double recombinant method in *X. dendrorhous*, whereby the deletion of *XdCrtI* in the homozygous strain causes a completely albino phenotype (Niklitschek et al., 2008). Here we determined that tp:XdCrtI:GFP with the pea small subunit RUBISCO transit peptide was correctly destined to plastids (Figure 3) and could be a suitable contender for enhancing total carotenoids and β-carotene when comparing pPSY2 and pPSY2-CRTI transgenic tomatoes (Figure 5C). The synthesis of carotenoids requires GGPP, a common precursor also needed for vitamin E (α-tocopherol), gibberellic acid and chlorophyll synthesis. Therefore, re-directing this precursor toward the synthesis of carotenoids by using constitutive promoters can have a negative impact on the metabolism of these other compounds. Consequently, a detrimental effect can be triggered both phenotypically and/or physiologically, with the generation of alterations such as dwarfism (Fray et al., 1995; Sandmann, 2001). A strategy to minimize this risk is to design genetic constructs with genes driven by tissue- or developmental-specific promoters. In this work, a 806 bp fragment of the fruit specific promoter of the tomato PG gene from *S. chilense* was chosen. This promoter has 99% identity to the 4,052–4,818 bp region of the *S. lycopersicum* PG promoter FJ465170.1 (Lau et al., 2009; Supplementary Figure 6), and targets specifically the transgenes to fruits (Figure 3 and Supplementary Figure 2). Although the expression of *PG* increases during apple ripening (Janssen et al., 2008), it has been established that the PG promoter does not depend on ethylene, and thus induces ripening in both climacteric fruits such as tomatoes (Lincoln et al., 1987), and non-climacteric fruit such as apples^7^. Importantly, the PG promoter from *S. chilense* is not subjected to intellectual property protection and is therefore available to be used without royalties. As a selection marker, we used *bar* that confers resistance to the BASTA (or phosphinothricin) herbicide. This gene is free of rights, and there are several currently commercialized transgenic plants^8^^,^^9^ harboring *bar*. The carotenogenic genes used here are also free of rights, even more so after optimizing their nucleotide sequence to make them more different from those native sequences present in carrot and yeast databases. For optimization, the sequence of interest was modified according to the preferential use of codons of the final organism, using the average of the codon usage of ribosomal genes or of a group of highly expressed genes (Puigbò et al., 2007). Here, the codon usage table of 567 mRNAs of *M. domestica* was available, and considered a sufficient population (163,086 codons) to successfully guide the codon usage preference, or at least to discriminate unusual codons (see text footnote 4). In the optimization of *DcPSY2*, *XdCrtI*, and *DcLCYB1*, most codons were conserved, since those used preferentially in carrots and yeast were the same as those in apples. However, there were codons that code for certain amino acids such as Tyr, Thr, Ser, Gln, Asn, Ile, Phe, and Glu where codon optimization was required.

### Carotenoid Increment in Tomato

Tomato represents a suitable model to evaluate our biotechnological platform designed for the functional assessment of candidate carotenogenic genes. The results obtained through this initial strategy allowed us, in a short period of time, to project possible results that were ultimately used to improve the carotenoid content in apples.

Mature tomato fruits accumulate significant amounts of the reddish lycopene, and very low levels of β-carotene and xanthophylls (Fraser and Bramley, 2004; Liu et al., 2015; D’Andrea and Rodriguez-Concepcion, 2019; Diretto et al., 2020). During ripening, carotenoid levels rise up to 400 times, of which lycopene represents 90% of the total, accumulating approximately 2,000 μg/g DW of this pigment (Fraser et al., 1994, 2002). This large accumulation of carotenoids is associated with several factors such as sink capacity and the activity of some plastidial proteins (Sun et al., 2018; D’Andrea and Rodriguez-Concepcion, 2019). Equally important is the regulatory role of the expression level of carotenogenic genes during ripening such as *PSY1* and *PDS* that present an increase whilst *LCYB* and *LCYE* decrease their expression during ripening (Giuliano et al., 1993; Pecker et al., 1996; Ronen et al., 1999, 2000).

As mentioned above, transgenic tomatoes expressing apple-optimized *DcPSY2*, *DcLCYB1*, and *XdCrtI* showed a dissimilar increase in the levels of total carotenoids, lycopene, β-carotene and lutein (Figure 5) which correlated only partially with gene expression (Figures 4f–i). Specifically, pPSY2 lines present up to 1.34-fold rises in total carotenoids and 1.25-fold increases in lycopene in P6 and P14 lines, whereas only P14 has a 2.5-fold increase in β-carotene, and P14 and P16 have a 1.27-fold increment in α-carotene (Figure 5A); P6 and P14 have the lowest *PSY2* expression level (Figure 4f). Nevertheless, this allows us to conclude that *DcPSY2* codes for a functional enzyme capable of increasing carotenoids in tomato fruits but at a moderate level and that a reduced expression level is sufficient to increment β-carotene (P14). Fraser et al., 2002 obtained an increase of up to 2.1 times in the total carotenoid content and lycopene in fruits of the Alisa Craig tomato variety transformed with the *CrtB* gene. This gene codifies a PSY from *E. uredovora*, which was expressed in a fruit-specific manner. These results suggest that DcPSY2 might not be as efficient as CrtB, but this should be confirmed by comparing the same variety of tomato.

Regarding pLCYB1 transgenic lines, they have almost two times more total carotenoids and lycopene and up to 2.5 times more β-carotene and 2.27 times more α-carotene in all transgenic lines (Figure 5B). Although L3 presents the lowest expression level, it is one of the two transgenic lines with the highest carotenoid content together with L6, the line with the highest expression level (Figures 4g, 5B). Previous reports have shown that the fruit-specific expression of *AtLCYB* in tomato var. Moneymaker (Rosati et al., 2000) and the constitutive expression of the endogenous *SlLCYB* in tomato var. Red Setter (D’Ambrosio et al., 2004) resulted in an increase of total carotenoid content of 1.6 times and 2 times, respectively, which is similar to the increase we obtained in pLCYB1 tomatoes var. Microtom (Figure 5). Regarding individual carotenoids, *AtLCYB* transgenic tomato fruits yielded on average 3.1 times more β-carotene than the control whereas the lycopene content remained constant (Rosati et al., 2000). Similarly, *SlLCYB* transgenic lines presented an increase of over ten times in β-carotene (D’Ambrosio et al., 2004); unlike Rosati et al., 2000, this was accompanied by a 53-fold drop in the lycopene levels (D’Ambrosio et al., 2004). Interestingly, *DcLCYB1* did not produce a detriment in lycopene content and actually produced an increase in α-carotene levels. The expression of *DcLCYB1* alone produces a twofold increment in total carotenoids (Figure 5B), similar to transplastomic tomatoes that expressed *LCYB* from *Narcissus pseudonarcissus* that produced a 50% increment in total carotenoids (Apel and Bock, 2009). Expressing *AtLCYB* or *EuCtrY* enabled the enhancement of β-carotene without substantial reductions in lycopene (Rosati et al., 2000; Wurbs et al., 2007). This shows that *DcLCYB1* is at least as equally efficient as *LCYB*s from other species. Indeed, tomatoes from pLCYB1 lines had a higher carotenoid content than tomatoes from the pPSY2 and pPSY2-CRTI lines (Figure 5), suggesting that in tomato the endogenous SlPSY, desaturases and isomerases are highly active. Our data revealed that the apple-optimized *XdCrtI* is functional in tomato. pPSY2-CRTI transgenic tomatoes exhibited a larger increase in total carotenoids and lycopene (twofold) in comparison to pPSY2 and also produced up to threefold more β-carotene and up to 1.75-fold more α-carotene compared to WT fruits (Figure 5C), especially in PC3 and PC4, the transgenic lines that have the lowest expression level of both *PSY2* and *CRTI*. This suggests that a low expression level is enough to increment the carotenoid level whilst a very high level of expression may not produce a significant increment and sometimes causes a detriment in total or individual carotenoid levels (Figure 5C). In previous studies, *EuCrtI* was constitutively expressed in tomato var. Alisa Craig which resulted in a twofold increase in β-carotene levels with a significant reduction in both total carotenoid content and lycopene, producing orange-colored fruits (Römer et al., 2000). This metabolic effect was also due to the induction of endogenous *PDS*, *ZDS*, and *LCYB* and a reduction in PSY1 enzyme activity (Römer et al., 2000). These results suggest that the pPSY2-CRTI construct is more efficient in terms of total carotenoids, lycopene, and lutein production than pPSY2.

The simultaneous expression of three carotenogenic genes in tomatoes has not been previously reported, although it is a strategy that was previously used in rice and potato (Ye et al., 2000; Diretto et al., 2007). Both in rice that simultaneously express *PSY*, *CRTI*, and *LCYB* (Ye et al., 2000) and in potato, that express *Erwinia* CrtB (*PSY*), CrtI, and CrtY (*LCYB*) (Diretto et al., 2007), there were increases in lutein and β-carotene contents in transgenic lines. On the contrary, in our study, the expression of pPSY2-CRTI-LCYB1 in tomatoes produces a significant decrease in total carotenoid content. This decrease correlates with 3.6-fold falls in lycopene and 7.3-fold falls in α-carotene, but not in β-carotene levels, which increased significantly (by 2.57-fold, Figure 5D). We propose that these alterations are responsible for the orange phenotype of these transgenic fruits (Figure 4e). The decrease in total carotenoids observed in the pPSY1-CRTI-LCYB1 lines, although it is an unexpected result, is similar to that obtained by Römer et al. (2000) in tomatoes expressing 35SCaMV:*CRTI*. Even though there are several reasons that could explain this phenomenon, one hypothesis is that the constitutive and high expression of *CRTI* in transgenic lines, increases the expression levels of *LCYB*, thus causing a concomitant increment in β-carotene synthesis (Römer et al., 2000). A second hypothesis is that the CRTI enzyme is more efficient, delivering either a more abundant or a more suitable substrate to the LCYB enzyme which then transforms it into β-carotene. These two possibilities are not mutually exclusive and actually together could have a synergistic effect increasing the β-carotene content (Giuliano et al., 2000). In our case, the simultaneous expression of the three key genes may be redirecting the metabolic flux of the carotenoid pathway toward the production of β-carotene at the expense of the α-carotene branch and of the accumulation of those that are synthesized in previous stages, producing fruits with the pale orange appearance. In addition, the dwarf *in vitro* phenotype of these lines can also be explained by the redirection of the metabolic flow of GGPP precursors of the MEP pathway to carotenoid synthesis, with a detriment of the synthesis of gibberellic acid (GA), causing negative effects on the general development and physiology of the plant. GGPP is a precursor shared for the synthesis of carotenoids and for that of several growth regulators such as cytokines, ABA and GA (Cuttriss et al., 2007; Howitt et al., 2007; Rodríguez-Concepción, 2010). GA is an important phytohormone involved in plant development (Yamaguchi, 2008). Tomato plants (var. Alisa Craig) that overexpress *PSY1* under the 35S promoter had fruits with higher levels of lycopene but presented a dwarf phenotype due to reduced levels of GA (Fray et al., 1995).

Taken together, our results allow us to suggest that pLCYB1 is the most suitable option for producing tomato fruits with a higher β-carotene and total carotenoid content.

### Carotenoid Increment in Apple Fruits

To evaluate our biotechnological platform, we chose a transient expression assay because it provides a rapid and robust tool for assessing the activity of genetic constructs before undertaking a stable transformation (Sparkes et al., 2006). This is especially relevant in non-model fruits such as apples (Arcos et al., 2020). However, the infiltration of apple fruits has inherent challenges, given their anatomical and physiological characteristics such as large parenchymatous cells with substantial vacuoles (Spolaore et al., 2001). Together with the stage of maturity of the fruits during the infiltration, these factors contribute to making agroinfiltration a highly heterogeneous and complex process (Suppplementary Figure 5). Nevertheless, once key steps of the procedure have been standardized, it is a feasible approximation for functional assessment, providing a valuable tool. Our data showed that in Fuji apple fruits, all four pCP-GC constructs were capable of modifying the content of total and individual carotenoids after transient agroinfiltration (Table 1). Indeed, the 1.17–2.27-fold increment in total carotenoids obtained with pPSY2-CRTI was similar to those values previously reported by Arcos et al. (2020) with the same genetic construct (2GC) but using a different transient transformation strategy (vacuum infiltration). Interestingly, data at 14-days post-infiltration showed that the total carotenoid content and β-carotene levels were in several cases up to twice and triple, respectively, to those obtained at 7-days post agroinfiltration (Table 1). This could be associated with the stability of the enzymes after infiltration as well as the storage mechanism of the pigments once produced. In addition, it shows that apple fruits were capable of continuing the normal ripening process despite the exogenous bacterial application, at least for the periods evaluated. This also allows us to conclude that *DcPSY2* is functional in apple fruits. Moreover, the increments seen in β-carotene also suggest that endogenous carotenogenic genes such as desaturases (PDS and ZDS), isomerases (Z-ISO and CRTISO) and LCYB are functional in apples.

The fact that the single constructs (pPSY2 and pLCYB1) duplicated the content of total carotenoids and β-carotene with respect to the double (pPSY2-CRTI) and triple constructs (pPSY2-CRTI-LCYB1) both at 7 and 14-days post infiltration, suggest that the transgenes in the double or triple constructs might not be properly expressed, or the stability of these transcripts/enzymes could be harmed. It is important to remember that each carotenogenic gene in the pCP constructs is driven by the same promoter, which could generate competition for the same transcription factors, limiting the recruitment of the transcription machinery, especially in the multiple constructs.

These results lead us to propose that apple fruits obtained from stable transformation will also accumulate at least two to three times more carotenoids. However, it is feasible to consider that the increment in the carotenoid levels would be even higher in the flesh considering our previous results, by expressing 35S:AtDXR in Fuji (Arcos et al., 2020). Specifically, leaves of AtDXR transgenic apples displayed up to a threefold rise in total carotenoids (508 mg g^–1^ FW) and in β-carotene (508 mg g^–1^ FW) when compared to wild-type plants, and twofold rises when transiently infiltrated in apple fruits (Arcos et al., 2020). In addition, in the flesh of banana fruits, a 2.3-fold increment in β-carotene was obtained by expressing *PSY1* or *PSY2* directed by a constitutive promoter when compared to the wild-type (Paul et al., 2017), and in kiwi fruit, a 1.3-fold increase in β-carotene was observed by expressing either *GGPPS* or *PSY* (Kim et al., 2010). Regarding the constructs generated, these results suggest that pPSY2 or pLCYB1 are the best candidates for the stable transformation of apples, and possibly for other fruits with pale flesh, in order to improve carotenoid content and thus nutritional properties.

## Conclusion

1. The PG promoter from *S. chilense* var. tomatillo correctly directs gene expression to fruits in tomato and apple.
2. *DcPSY2* codifies for a functional enzyme with a plastidial localization.
3. All four pCP-CG vectors are able to increase the total carotenoid content, particularly of β-carotene, in tomatoes and apples.
4. Of the pCP-CG vectors, those that possess the carrot carotenogenic genes *DcPSY2* and *DcLCYB1*, would be the best candidates for increasing carotenoids in stably transformed apples.

## Data Availability Statement

The original contributions presented in the study are included in the article/Supplementary Material, further inquiries can be directed to the corresponding author/s.

## Author Contributions

AA-M and CS conceived and designed the experiments. DA, AA-M, CF-O, and CP performed the experiments. DA, AA-M, CF-O, MH, and CS wrote the manuscript. All authors contributed to the article and approved the submitted version.

## Conflict of Interest

The authors declare that the research was conducted in the absence of any commercial or financial relationships that could be construed as a potential conflict of interest.

## Publisher’s Note

All claims expressed in this article are solely those of the authors and do not necessarily represent those of their affiliated organizations, or those of the publisher, the editors and the reviewers. Any product that may be evaluated in this article, or claim that may be made by its manufacturer, is not guaranteed or endorsed by the publisher.

## Footnotes

1
http://faostat.fao.org

2
http://www.genscript.com

3
http://genomes.urv.es/OPTIMIZER/

4
http://www.kazusa.or.jp

5
http://linux1.softberry.com

6
http://www.ncbi.nlm.nih.gov/nuccore/V00087

7
http://www.postharvest.com

8
www.isaa.org

9
www.chilebio.cl
