# Supplementary material for: Daucus carota DcPSY2 and DcLCYB1 as Tools for Carotenoid Metabolic Engineering to Improve the Nutritional Value of Fruits
Source: Front Plant Sci. 2021 Aug 26;12:677553. doi: 10.3389/fpls.2021.677553 (PMC8427143; doi:10.3389/fpls.2021.677553)
Supplement: Supplementary file 1 [file Data_Sheet_1.docx]

**Supp Table 1: Primers used in this work.**

| **Primer ID** | **Primer sequence** | **Fragment length (bp)** | **Description** |
| --- | --- | --- | --- |
| Oligo AP | 5’ CGCCACGCGTCGACTAGTACTTTTTTTTTTTTTTTTT 3’ | - | For cDNA synthesis |
| PSY2opt.R | 5’ CAATCCAACGGTTCCAGC 3’ | 606 | RT-PCR of transgenic fruits |
| PSY2opt.F | 5´CTTGGGAGGAAGCGAGTTGGAG 3’ |  |  |
| LCYB1opt.R | 5’ CCTTAATTCCCAAGTGTCTCAATCTAGCA 3’ | 1000 | RT-PCR of transgenic fruits |
| LCYB1opt.F | 5` ATGAAGGTTATGGATACCTTGTTGAAGAC 3’ |  |  |
| CRTIopt.R | 5` GCTTGAACAAATCTGGCAAC 3’ | 310 | RT-PCR of transgenic fruits |
| CRTIopt.F | 5` CCCTTCCCATACCTCTTATC 3’ |  |  |
| SlPSY.R | 5’ TTATCTTTGAAGAGAGGCAGTTT 3’ | 1200 |  |
| SlPSY.F | 5’ TTATCTTTGAAGAGAGGCAGTTT 3’ |  |  |
| pPG F | 5´GTACCATCACATAATTGAGACG 3´ |  |  |
| PSY2opt.R ws | 5´GGTCTTCAAGAAGGTGCTGC 3´ | 2124 | Together with pPG F was used to amplify pPG-PSY2 without the stop codon |
| CRTIopt R ws | 5´GAAAGCCAAAACTCCAACGC 3´ | 2719 | Together with pPG F was used to amplify pPG-tpCRTI without the stop codon |
| qActin. R | 5’ CAGTTAAATCACGACCAGCAAGAT 3’ |  |  |
| qActin.F | 5’ TGTCCCTATTTACGAGGGTTATGC 3’ |  |  |
| qPSYopt. R | 5’ AACCATGCTGCTGTGAACTG 3’ |  |  |
| qPSYopt.F | 5’ GGAGGAAGATTGGAGAAGGG 3’ |  |  |
| qCRTIopt. R | 5’ GTG TCT GGT GCT TGG TCT GA 3’ |  |  |
| qCRTIopt. F | 5’ TGC TGA GAA GGT TCA CGA TG 3’ |  |  |
| qLCYBopt.F | 5’ GGG AGG ACC ATT GCC AGT TTT 3’ |  |  |
| qLCYBopt.R | 5’ GGA GCA GCA GCC AAG GTT CTA 3’ |  |  |


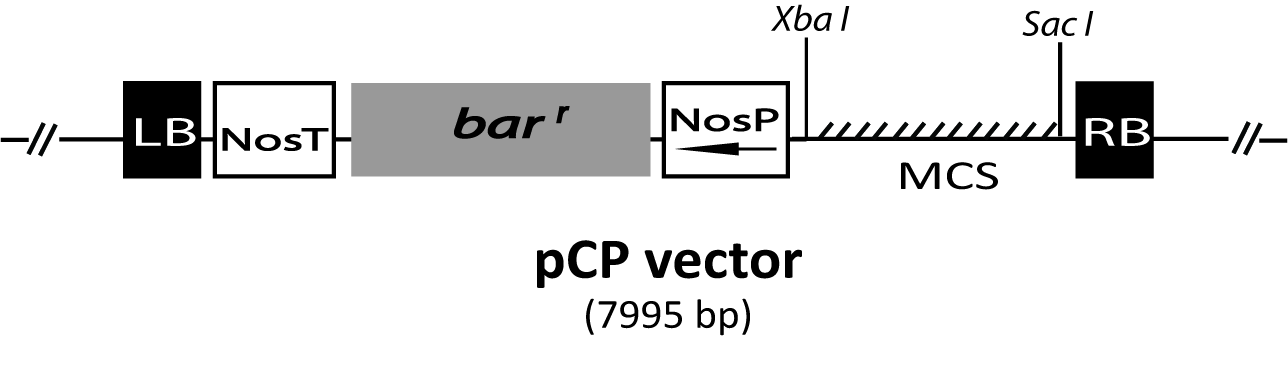


**Sup Figure 1. Diagram of the pCP vector.** The pCP vector was generated using a 7,854 bp backbone from pB7FWG2.0 including the Right and Left Border (RB and LB), and Basta (bar^r^) as a resistance marker for transgenic plant selection. A specially designed Multiple Cloning Site (MCS) of 141 bp was cloned between the XbaI and SacI restriction sites. MCS: 5' - EcoRI, ApaI, PstI, AvrII, EcoRV, BspEI, NcoI, BglII - 3'.


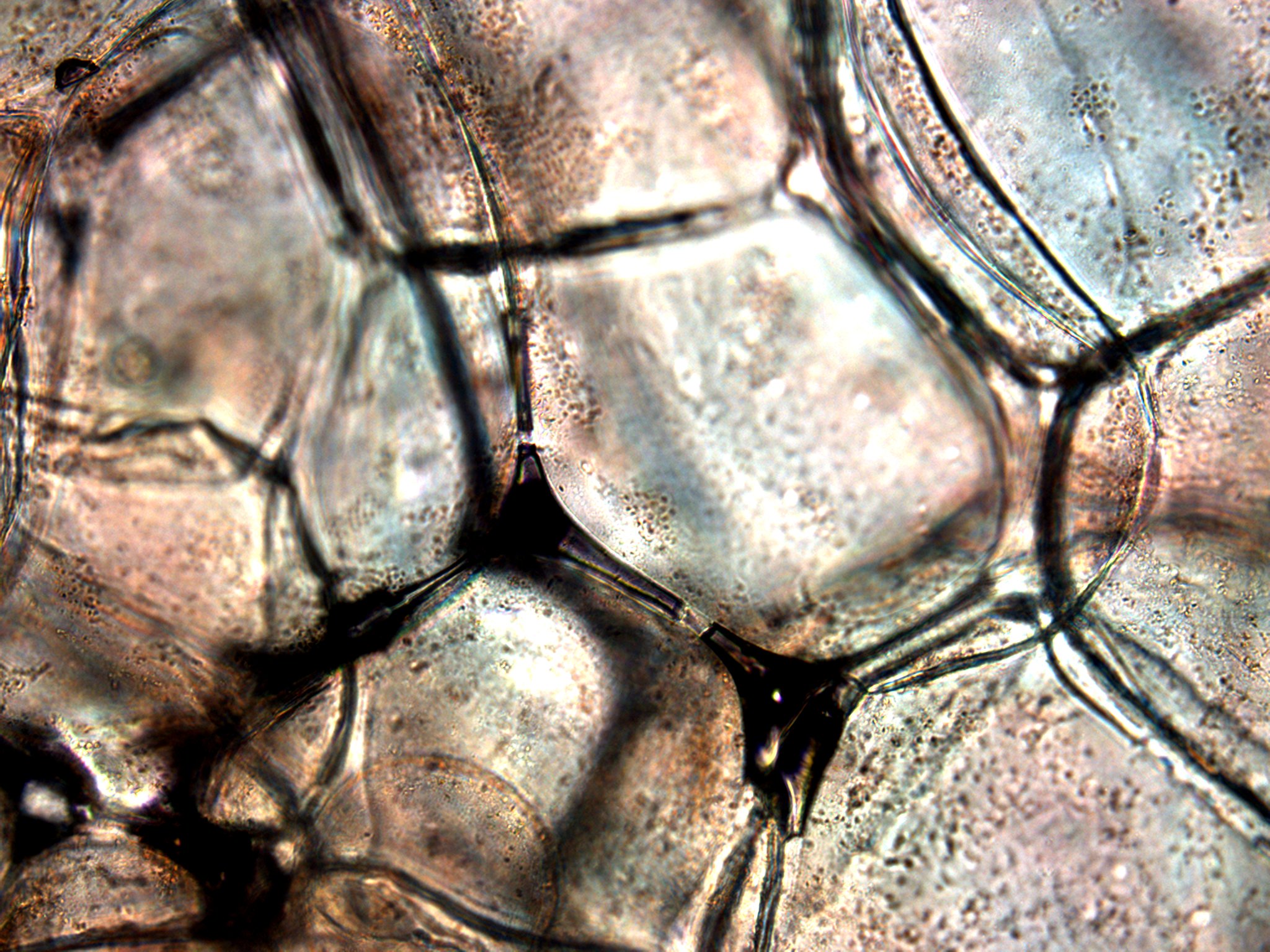

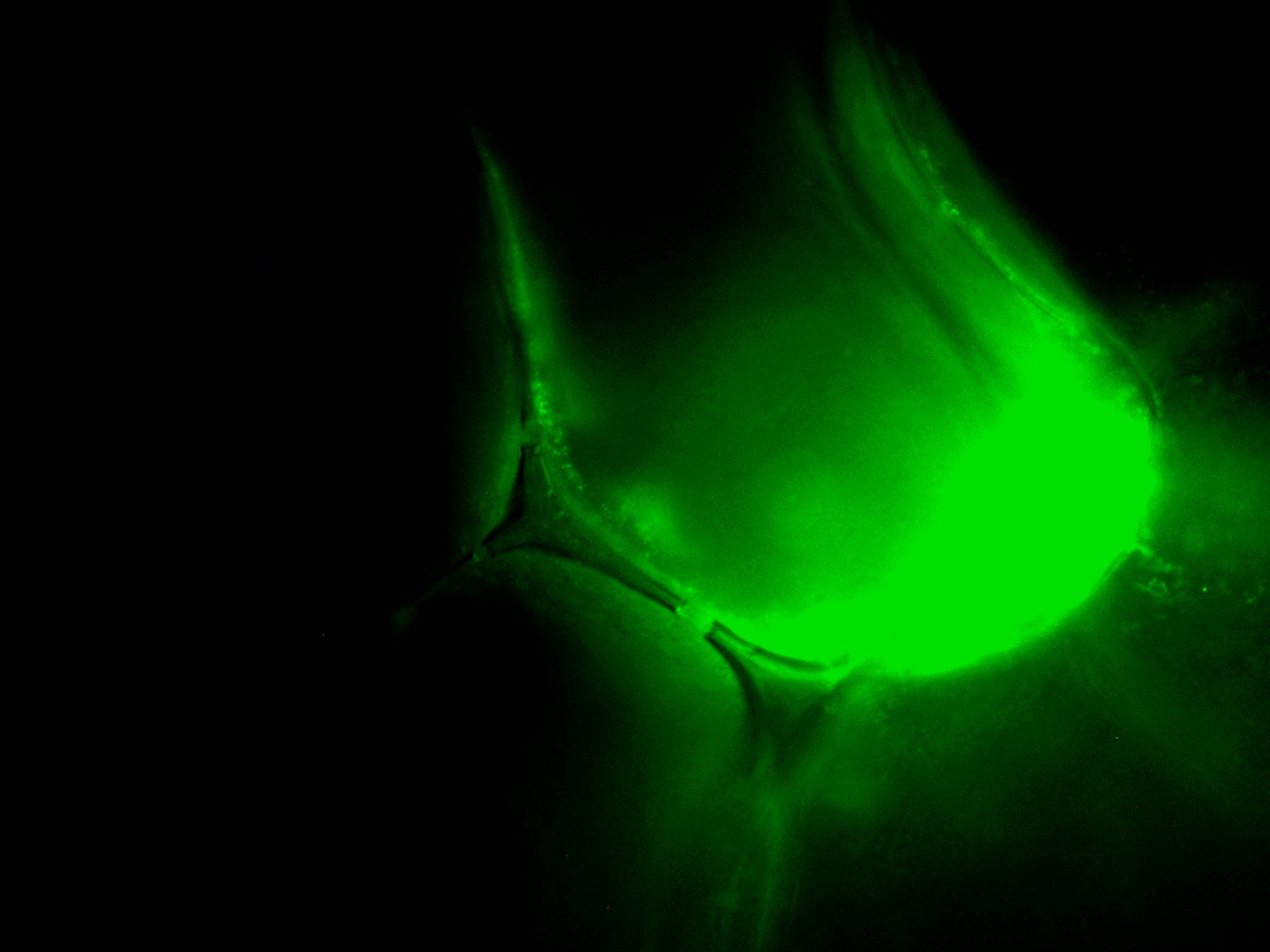


a


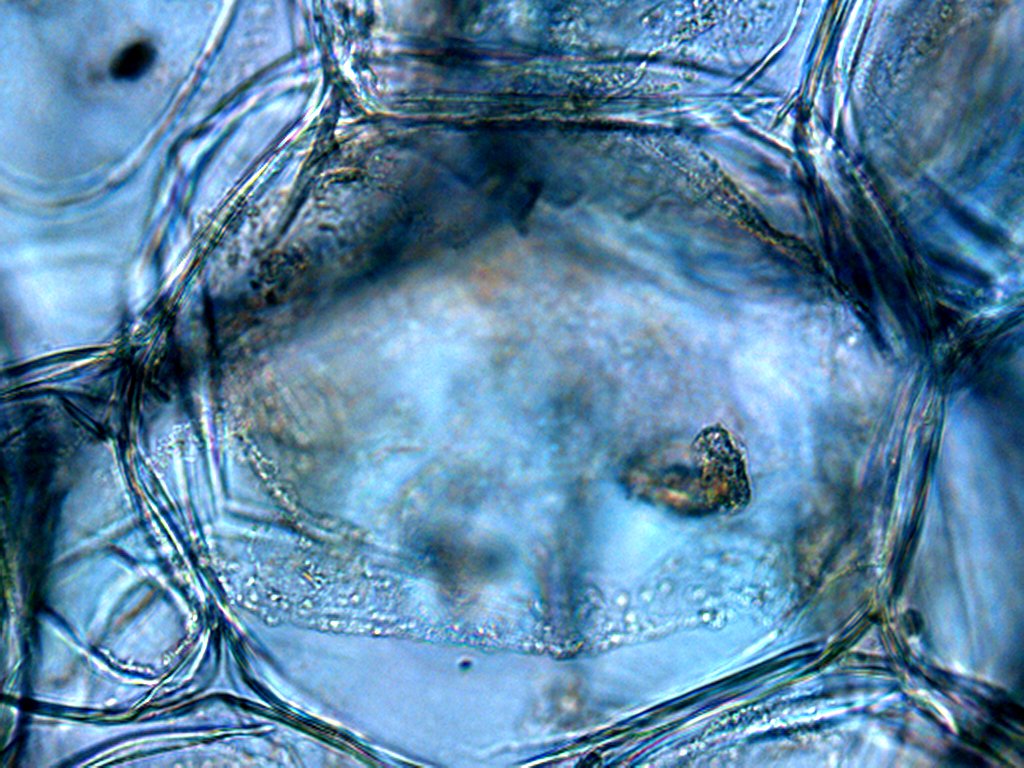

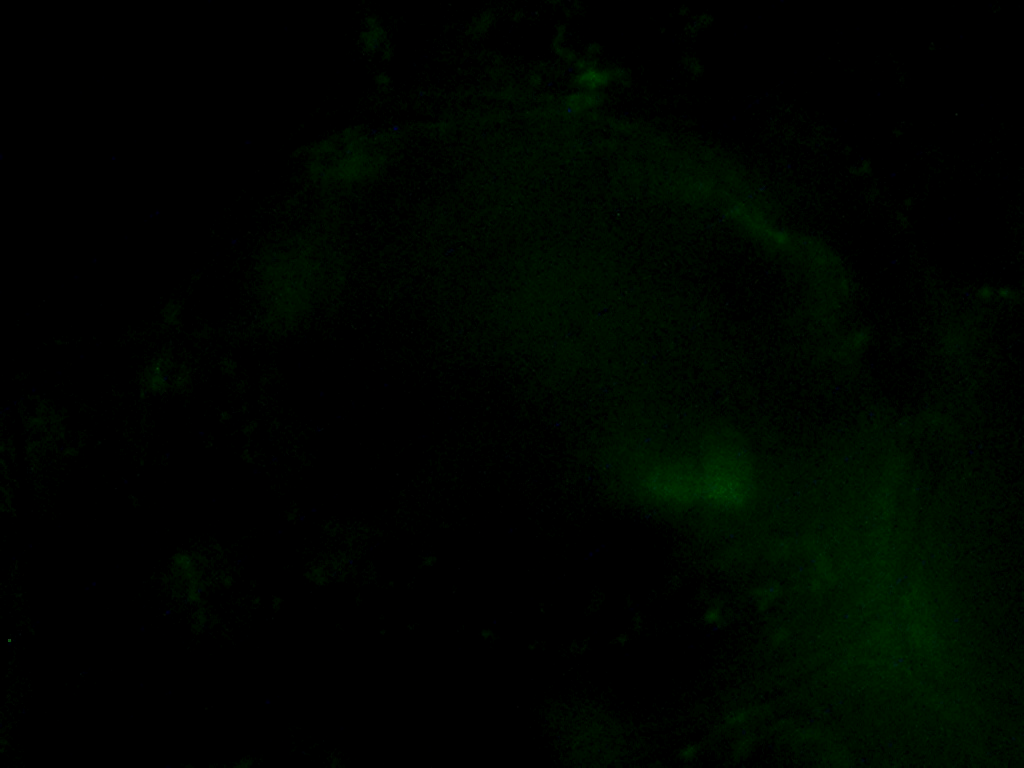


b

c

d

pPG:GFP

Empty Vector


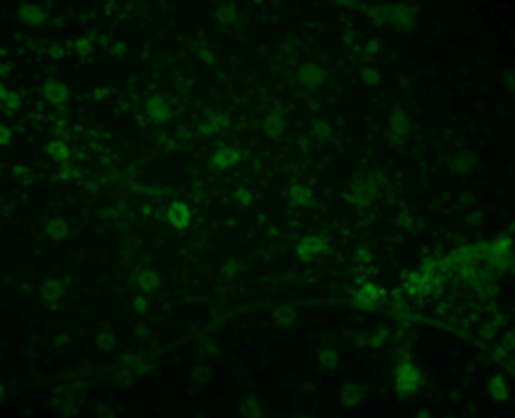

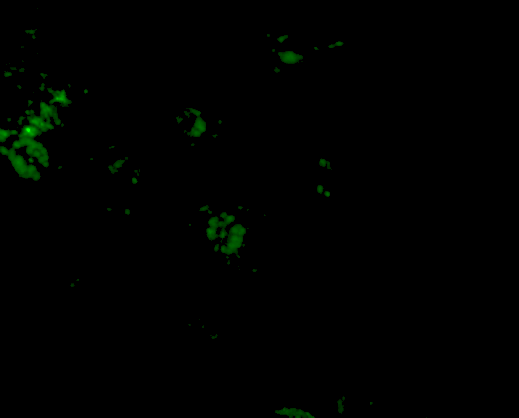


pPG:GFP

e

f

**
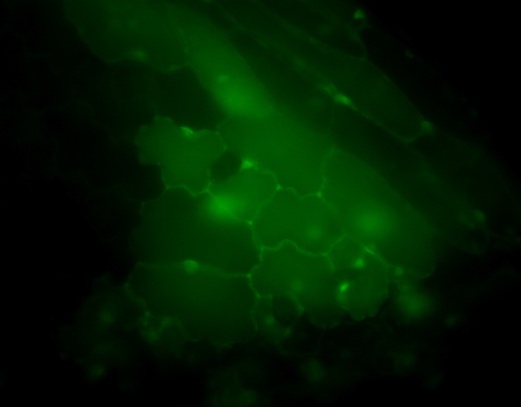
**

g

35SCaMV:GFP

**Supp. Figure 2. Control of GFP emission.** a: cytoplasmatic localization of soluble GFP driven by the PG promoter (pPG:GFP construct) in transiently transformed tomato fruits. b: Bright field of a. c: GFP background fluorescence in tomato fruits transiently transformed with the empty vector pMDC107, which includes the PG promoter with no gene cloned downstream. d: Bright field of c. e: pPG:GFP construct transiently infiltrated in tobacco leaves. f: Uninfiltrated tobacco leaves. G: 35SCaMV:GFP construct transiently infiltrated in tobacco leaves. The autofluorescence of chlorophyll is observed in e and f . GFP fluorescence was observed at 465-495 nm excitation in the other pannels. Scale bars: Panel a-d: 50 µm, panel e-f: 100 μm.


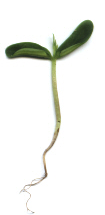

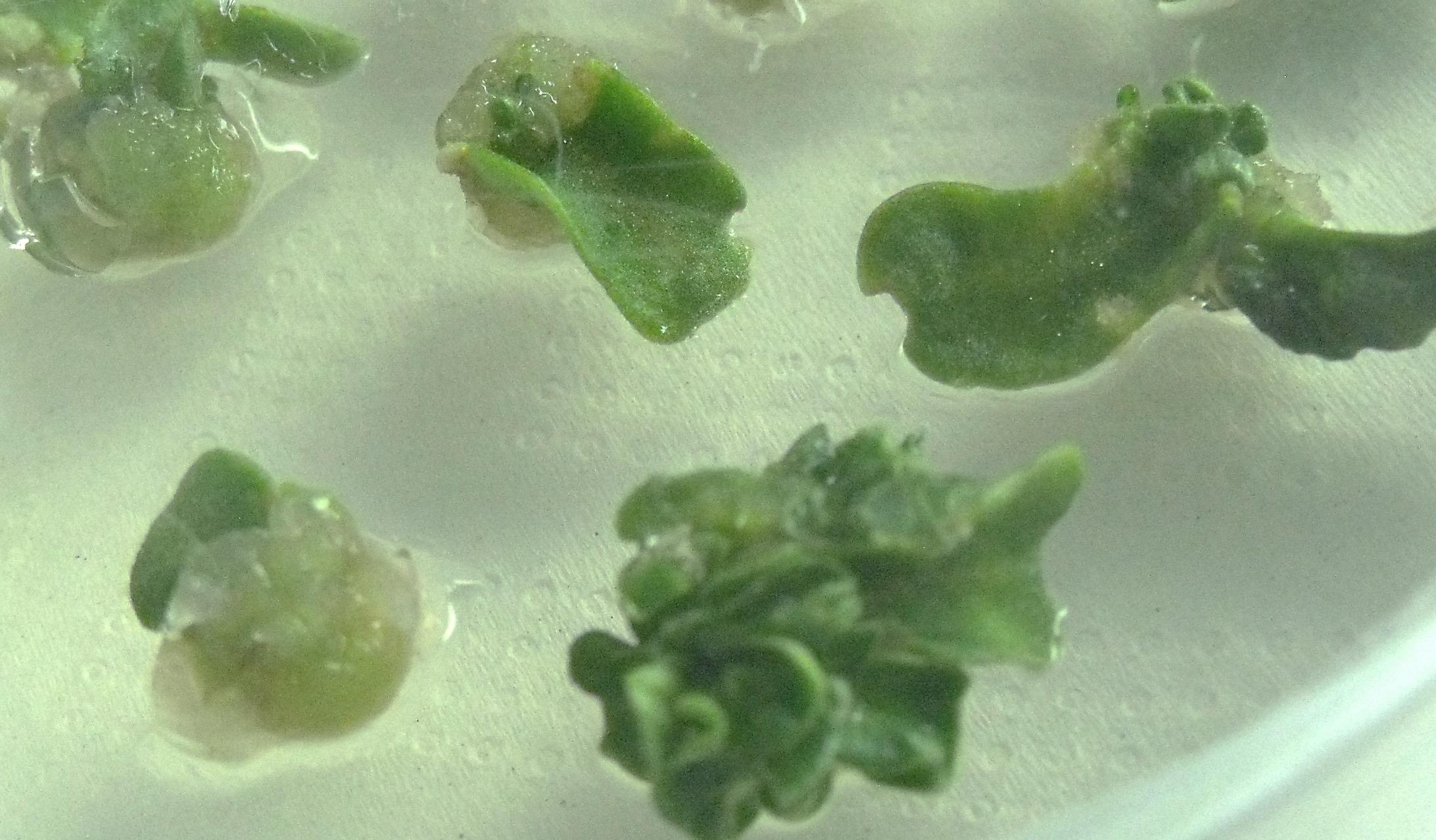

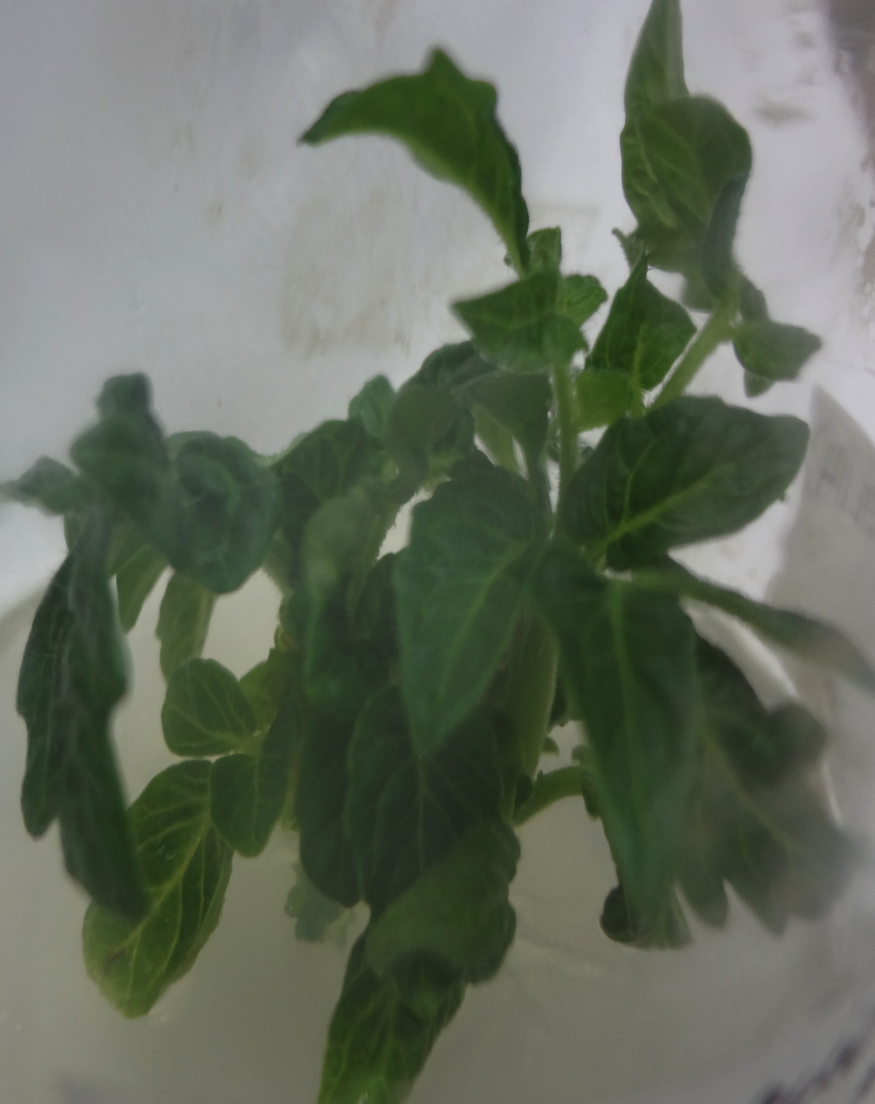

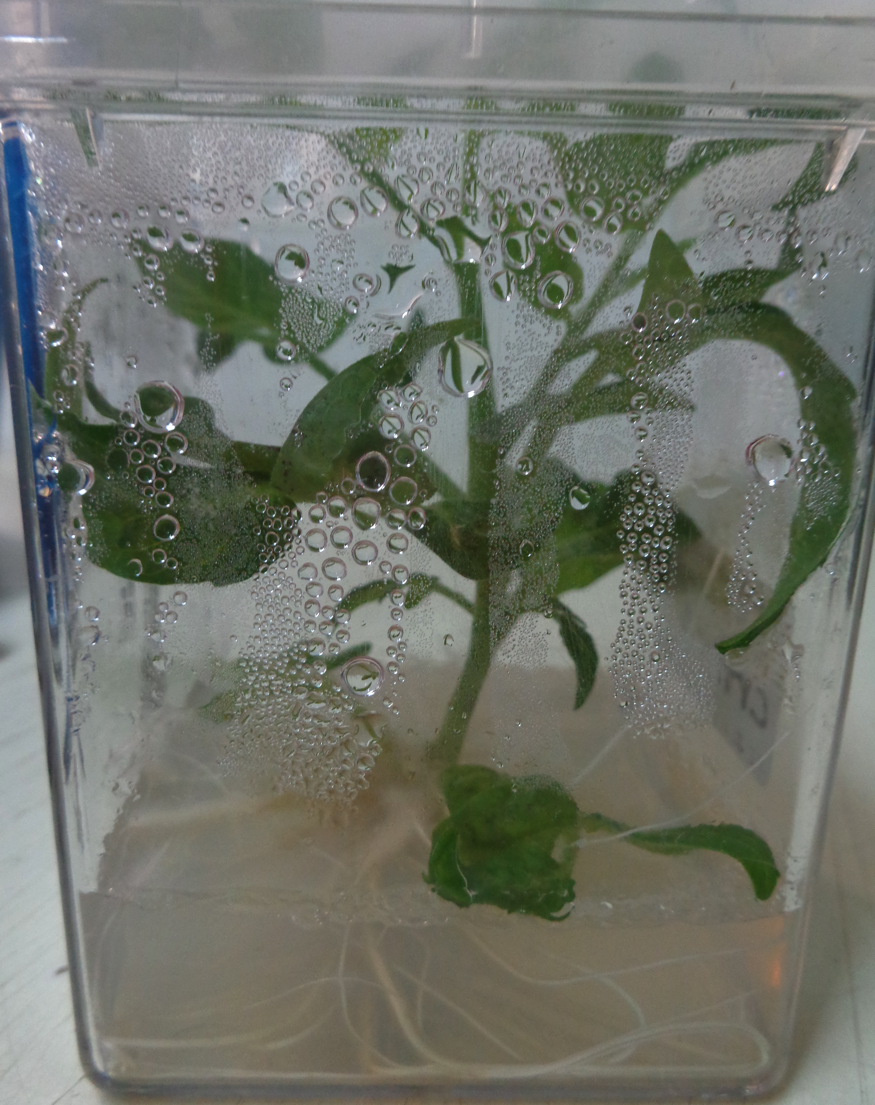

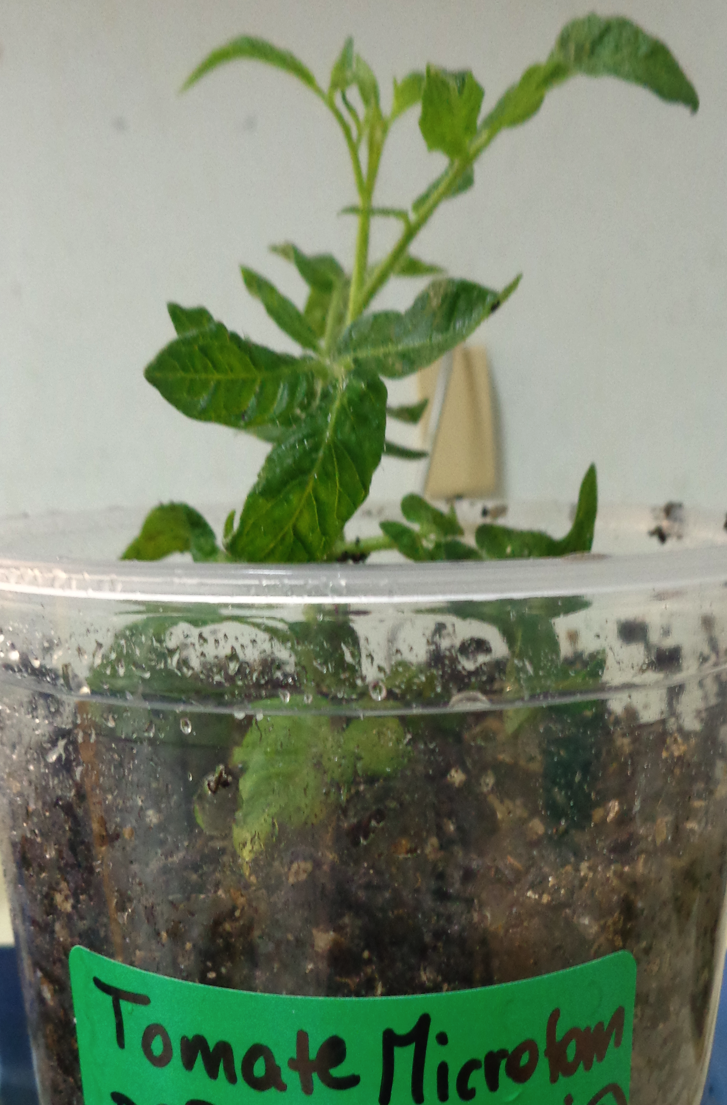

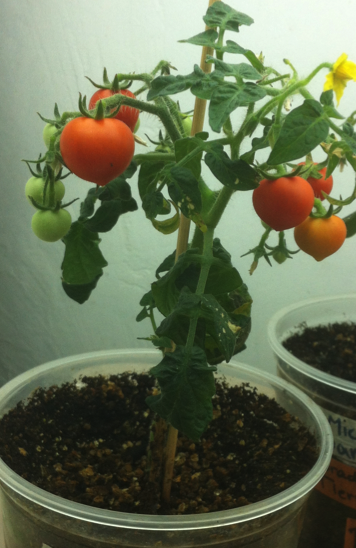


a

b

c

d

e

f


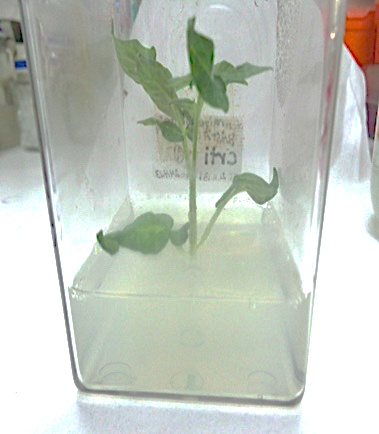

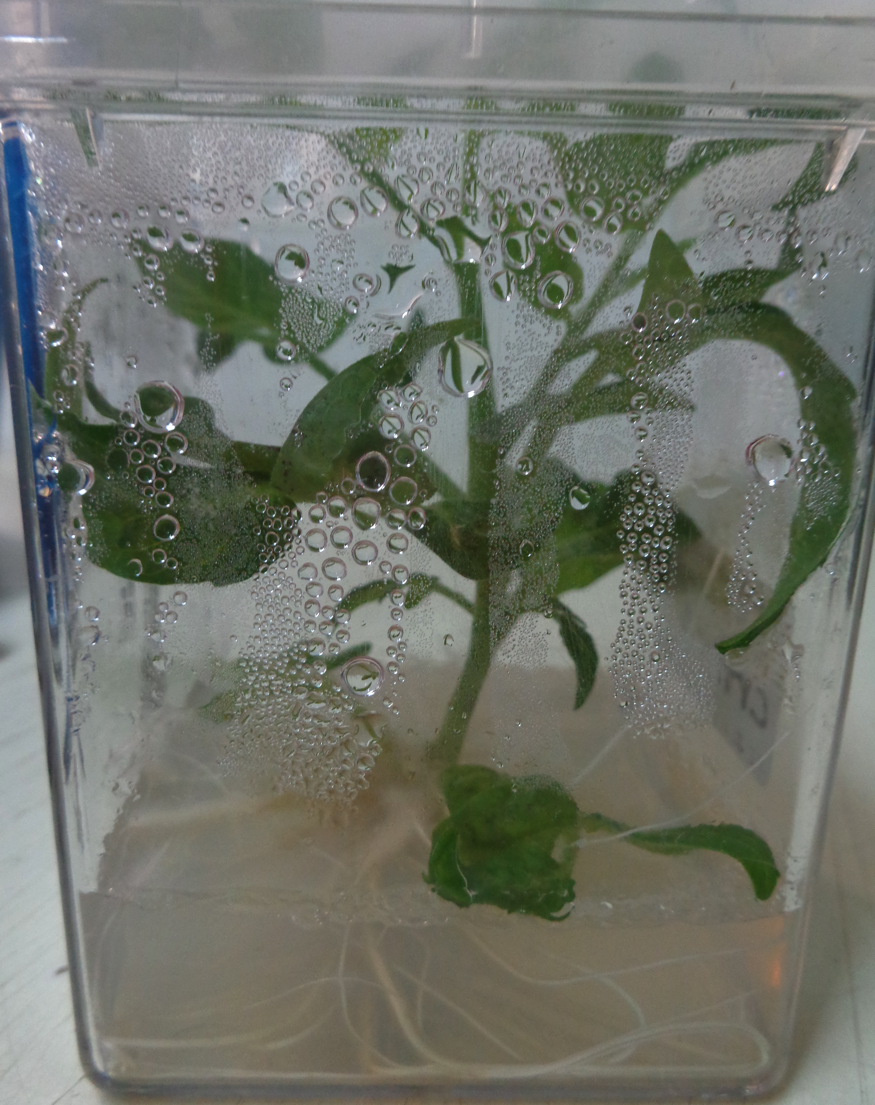

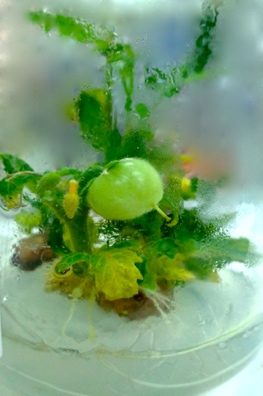


h

i

j

g


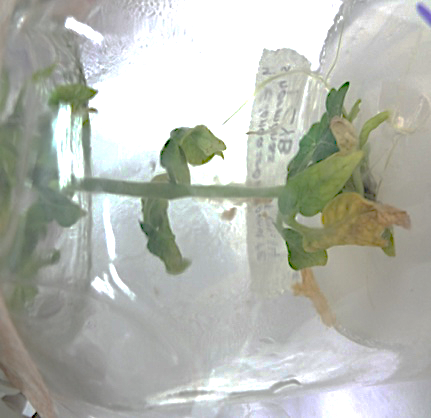


**Supp. Figure 3: Stable transformation of tomatoes (cv. Microtom)** (a) Leaves from two weeks-old in vitro seedlings were incubated with Agrobacterium for two days. Explants were then transferred and maintained in Induction medium with antibiotics for selection and Agrobacterium inhibition. Scale bar = 10 mm. (b) Calli induction (black arrow) and development of an initial shoot (white arrow) were triggered in the Induction media. Scale bar = 10 mm. (c) Shoots were cultivated in Elongation media for vegetative development and shoot growth until plant material reached 3-5 cm length with 4-5 leaves. Scale bar = 1 cm. (d) Shoots developed a radicular system when transferred into Rooting media. Scale bar = 2 cm. (e) Plants were transferred to soil and acclimated to greenhouse conditions. Scale bar = 3 cm. (f) Plants reached maturity and started flower development and fruit production. Scale bar = 3 cm. g) same as in d and corresponds to pPSY2, h) corresponds to pPSY2-CRTI, i) corresponds to pLCYB and j) corresponds to pPSY2-CRTI-LCYB before acclimation.


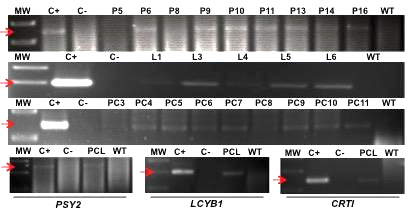


**pPSY2**

**a**

**pLCYB1**

**b**

**pPSY2-CRTI**

**c**

**pPSY2-CRTI-LCYB1**

**d**


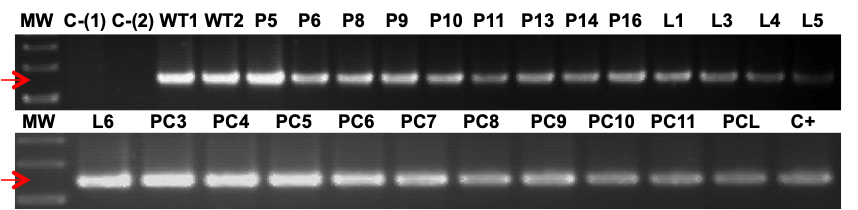


***SlPSY***

**e**

***SlPSY***

**Supp. Figure 4: RT-PCR of transgenic tomato plants.** Expression of transgenes from transgenic lines of *S. lycopersicum* Microtom. a) A 606 bp fragment of *DcPSY2* was amplified from pPSY2 lines. b) A 1 kb fragment of *DcLCYB1* was amplified from pLCYB1 lines. c) a 310 bp fragment of *XdCrtI* was amplified from pPSY2-CRTI lines. d) a 606 bp fragment of *DcPSY2*, a 1 kb fragment of *DcLCYB1* and a 310 bp fragment of *XdCrtI* were amplified from the pooled fruits from pPSY2-CRTI-LCYB1 lines. e) a 1.2 kb fragment of endogenous *SlPSY1* was amplified from each of the above mentioned lines showing cDNA integrity (positive control). MW: molecular weight markers (1 kb), C+: the corresponding fragment was amplified from the pCP vector, C-: RT-PCR without cDNA, WT: wild type cDNA. Red arrows indicate the amplicons of interest.





**Supp. Figure 5: Transitory agroinfiltration and carotenoid quantification in apple fruits.** As part of the standardization of transitory agroinfiltration protocols, we evaluated the total carotenoid content in Fuji and Granny Smith apple fruits after one week of agroinfiltration with a pCP-CG construction using the agroinjection system. a) Representative Fuji apples (upper panels) and Granny Smith apples (lower panels) seven days post-agroinfiltration with the vector pPSY2-CRTI-LCYB1 and the WT non-agroinfiltrated. The right pictures showed the agroinfiltrated area (red square), which displayed a characteristic oxidation phenotype in the agroinfiltrated area. b) Graphical quantification of total carotenoids seven days post-agroinfiltration in Fuji and Granny Smith showed significant increments in the total carotenoids content in the two apple varieties agroinfiltrated with respect to the WT areas. Carotenoid content was expressed in μg/gFW (fresh weight) The columns represent the means and standard deviation of at least three biological replicas. P < 0.05 (*), P < 0.001 (***).


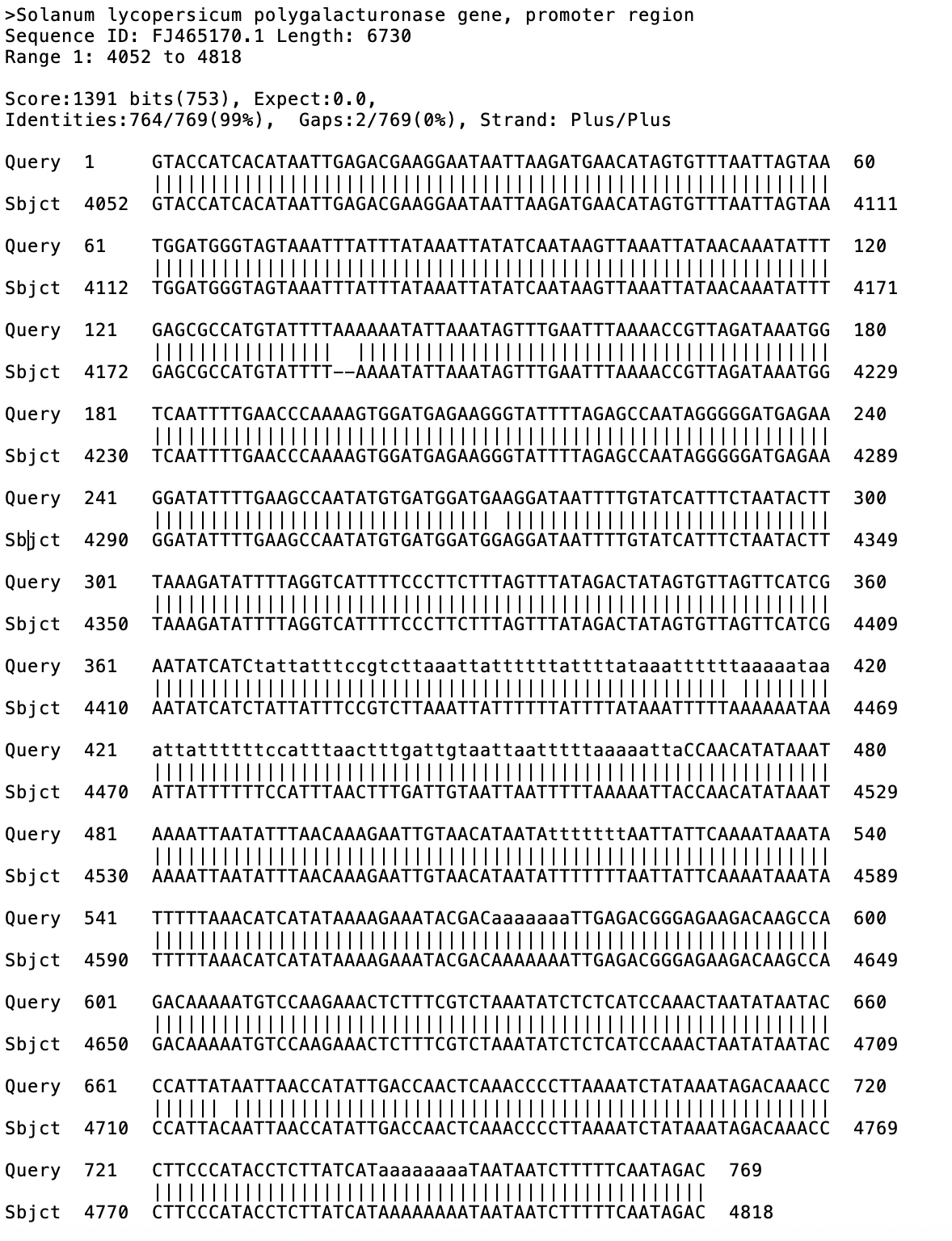


**Sup Figure 6: Alignment between *S. chilensis* and *S. lycopersicum* PG promoter.** Query: PG promoter amplified from S. chilensis, Sbjct: *S. lycopersicum* promoter FJ465170.1 of 6730 length that aligned 99% between 4052-4818

**Supplemental Material**

*DcPSY2* (DQ192187) optimized for *Malus domestica*.

ATGAGCGTTGCTATGAGCTGGATTGTTACCCCAAGCTTGGAGGTTAGCAACTGCTTCGGA

TACTTGGAGACCGCTAGAGAGGGAACCAGAGTTTTGGATCCAAGCAGATTGGGAAGCAGA

GATAAGAACATGAGATGCGGAGGAAGATTGGAGAAGGGAAAGTTGAGAAAGTGGAGCAGC

AAGAGCTTCAACGCTGAGTACAGCTACAGCTGCTTGGGAGGAAGCGAGTTGGAGAACGGA

AGCATTTTCCCAGTTCACAGCAGCATGGTTGTTAGCGCTGATGGAGATATGGCTGTTAGC

AGCGAGAAGAAGGTTTACGATGTTGTTTTGAAGCAGGCTGCTTTGGTTAAGAGACAGTTC

AGAAGCGATGAGGAGTTGGAGGTTAAGCCAGAGATGATTTTGCCAGGAACCTTGAGCTTG

TTGAGCGAGGCTTACGATAGATGCGGAGAGGTTTGCGCTGAGTACGCTAAGACCTTCTAC

TTGGGAACCTTGTTGATGACCCCAGAGAGAAGAAGAGCTATTTGGGCTATTTACGTTTGG

TGCAGAAGAACCGATGAGTTGGTTGATGGACCAAACGCTAGCCACATTACCCCAAGCGCT

TTGGATAGATGGGAGTTGAGATTGGAGGATTTGTTCAAGGGAAGACCATTCGATATGTTG

GATGCTGCTTTGAGCGATACCGTTATGAAGTTCCCAGTTGATATTCAGCCATTCAAGGAT

ATGATTGAGGGAATGAGAATGGATTTGAAGAAGAGCAGATACAAGAACTTCGATGAGTTG

TACTTGTACTGCTACTACGTTGCTGGAACCGTTGGATTGATGAGCGTTCCAATTATGGGA

ATTGCTCCAAACAGCCAGGCTACCACCGAGAGCGTTTACAACGCTGCTTTGGCTTTGGGA

TTGGCTAACCAGTTGACCAACATTTTGAGAGATGTTGGAGAGGATGCTAGAAGAGGAAGA

GTTTACTTGCCACAGGATGAGTTGGCTCAGGCTGGATTGAGCGATGAGGATATTTTCGCT

GGAAAGGTTACCGATAAGTGGAGAAACTTCATGAAGAAGCAGATTAAGAGAGCTAGAATG

TTCTTCGATGAGGCTCAGATTGGAGTTAGAGAGTTGAGCCCAGCTAGCAGATGGCCAGTT

TGGGCTAGCTTGTTGTTGTACAGACAGATTTTGGATGAGATTGAGGCTAACGATTACAAC

AACTTCACCAAGAGAGCTTACGTTAGCAAGCCAAAGAAGATTTTGGCTTTGCCAGTTGCT

TACGCTAAGGCTTTCGCTCCAACCGCTAGAACCAGCAGCACCTTCTTGAAGACCTGA

CrtI (gi.68250374) optimized for *Malus domestica*

ATGGGAAAGGAGCAGGATCAGGATAAGCCAACCGCTATTATTGTTGGATGCGGAATTGGA

GGAATTGCTACCGCTGCTAGATTGGCTAAGGAGGGATTCCAGGTTACCGTTTTCGAGAAG

AACGATTACAGCGGAGGAAGATGCAGCTTGATTGAGAGAGATGGATACAGATTCGATCAG

GGACCAAGCTTGTTGTTGTTGCCAGATTTGTTCAAGCAGACCTTCGAGGATTTGGGAGAG

AAGATGGAGGATTGGGTTGATTTGATTAAGTGCGAGCCAAACTACGTTTGCCACTTCCAC

GATGAGGAGACCTTCACCTTGAGCACCGATATGGCTTTGTTGAAGAGAGAGGTTGAGAGA

TTCGAGGGAAAGGATGGATTCGATAGATTCTTGAGCTTCATTCAGGAGGCTCACAGACAC

TACGAGTTGGCTGTTGTTCACGTTTTGCAGAAGAACTTCCCAGGATTCGCTGCTTTCTTG

AGATTGCAGTTCATTGGACAGATTTTGGCTTTGCACCCATTCGAGAGCATTTGGACCAGA

GTTTGCAGATACTTCAAGACCGATAGATTGAGAAGAGTTTTCAGCTTCGCTGTTATGTAC

ATGGGACAGAGCCCATACAGCGCTCCAGGAACCTACAGCTTGTTGCAGTACACCGAGTTG

ACCGAGGGAATTTGGTACCCAAGAGGAGGATTCTGGCAGGTTCCAAACACCTTGTTGCAG

ATTGTTAAGAGAAACAACCCAAGCGCTAAGTTCAACTTCAACGCTCCAGTTAGCCAGGTT

TTGTTGAGCCCAGCTAAGGATAGAGCTACCGGAGTTAGATTGGAGAGCGGAGAGGAGCAC

CACGCTGATGTTGTTATTGTTAACGCTGATTTGGTTTACGCTAGCGAGCACTTGATTCCA

GATGATGCTAGAAACAAGATTGGACAGTTGGGAGAGGTTAAGAGAAGCTGGTGGGCTGAT

TTGGTTGGAGGAAAGAAGTTGAAGGGAAGCTGCAGCAGCTTGAGCTTCTACTGGAGCATG

GATAGAATTGTTGATGGATTGGGAGGACACAACATTTTCTTGGCTGAGGATTTCAAGGGA

AGCTTCGATACCATTTTCGAGGAGTTGGGATTGCCAGCTGATCCAAGCTTCTACGTTAAC

GTTCCAAGCAGAATTGATCCAAGCGCTGCTCCAGAGGGAAAGGATGCTATTGTTATTTTG

GTTCCATGCGGACACATTGATGCTAGCAACCCACAGGATTACAACAAGTTGGTTGCTAGA

GCTAGAAAGTTCGTTATTCACACCTTGAGCGCTAAGTTGGGATTGCCAGATTTCGAGAAG

ATGATTGTTGCTGAGAAGGTTCACGATGCTCCAAGCTGGGAGAAGGAGTTCAACTTGAAG

GATGGAAGCATTTTGGGATTGGCTCACAACTTCATGCAGGTTTTGGGATTCAGACCAAGC

ACCAGACACCCAAAGTACGATAAGTTGTTCTTCGTTGGAGCTAGCACCCACCCAGGAACC

GGAGTTCCAATTGTTTTGGCTGGAGCTAAGTTGACCGCTAACCAGGTTTTGGAGAGCTTC

GATAGAAGCCCAGCTCCAGATCCAAACATGAGCTTGAGCGTTCCATACGGAAAGCCATTG

AAGAGCAACGGAACCGGAATTGATAGCCAGGTTCAGTTGAAGTTCATGGATTTGGAGAGA

TGGGTTTACTTGTTGGTTTTGTTGATTGGAGCTGTTATTGCTAGAAGCGTTGGAGTTTTG

GCTTTCTGA

*LCYB1* (DQ192190) optimized for *Malus domestica*

ATGAAGGTTATGGATACCTTGTTGAAGACCCACAACAAGTTGGAGTTCTTCAACCCAATT

CACGGATTCCCAGATAAGGTTGGAACCTTGAGCTGCTTGAAGTTCAGAAACCAGGAGTTG

AGATTCGGAAGCAGAAGAAGCAACGTTAACTGGGGAAAGAACGGAAGCGTTAAGGCTAGC

AGCAGCGCTTTGTTGGAGTTGGTTCAGGAGACCAAGAAGGAGAACTTGGAGTTCGATTTG

CCATTGTACGATCCAAGCAACGGATTGGTTGTTGATTTGGCTGTTGTTGGAGGAGGACCA

GCTGGATTGGCTGTTGCTCAGCAGGTTAGCGAGGCTGGATTGGCTGTTGTTAGCATTGAT

CCAAGCCCAAAGTTGATTTGGCCAAACAACTACGGAGTTTGGGTTGATGAGTTCGAGGCT

ATGGATTTGTTGGATTGCTTGGATACCACCTGGAGCAGCGCTATTGTTTACATTGATGAT

CAGACCACCAAGGAGTTGGGAAGACCATACGGAAGAGTTAACAGAAAGCAGTTGAAGAGC

AAGATGATGCAGAAGTGCATTAGCAACGGAGTTAAGTTCCACCAGGCTAAGGTTGTTAAG

GTTGTTCACGAGGAGGCTAAGAGCTTGTTGATTTGCGATGATGGAGTTACCATTCAGGCT

GCTGTTGTTTTGGATGCTACCGGATTCAGCAGATGCTTGGTTCAGTACGATAAGCCATAC

AACCCAGGATACCAGGTTGCTTACGGAATTGTTGCTGAGGTTGAGGAGCACCCATTCGAT

GTTAACAAGATGATTTTCATGGATTGGAGAGATAGCCACTTGAACGGAAACACCGAGTTG

AAGGAGAGAAACAGCAAGATTCCAACCTTCTTGTACGCTATGCCATTCAGCAGCGATAGA

ATTTTCTTGGAGGAGACCAGCTTGGTTGCTAGACCAGGATTGGCTATGGGAGATATTCAG

GAGAGAATGGTTGCTAGATTGAGACACTTGGGAATTAAGGTTAAGAGCATTGAGGAGGAT

GAGAGATGCGTTATTCCAATGGGAGGACCATTGCCAGTTTTGCCACAGAGAGTTGTTGGA

ATTGGAGGAACCGCTGGAATGGTTCACCCAAGCACCGGATACATGGTTGCTAGAACCTTG

GCTGCTGCTCCAGTTGTTGCTAACGCTATTGTTCAGTACTTGGGAGGAAGCAAGAAGGGA

GCTTTGGGAAACGAGTTGAGCGCTGAGGTTTGGAAGGATTTGTGGCCAATTGAGAGAAGA

AGACAGAGAGAGTTCTTCTGCTTCGGAATGGATATTTTGTTGAAGTTGGATTTGCCAGGA

ACCAGAAGATTCTTCAGCGCTTTCTTCGATTTGGAGCCAAGATACTGGCACGGATTCTTG

AGCAGCAGATTGTTCTTGCCAGAGTTGTTCTTCTTCGGATTGAGCTTGTTCAGCAACGCT

AGCAACACCAGCAGAATTGAGATTATGGCTAAGGGAACCGTTCCATTGGTTAACATGGTT

AACAACTTGATTAAGGATAGAGAGTAG

EC 4.1.1.39 *Pisum sativum* (pea) ribulose bisphosphate carboxylase small subunit: Location:1..171 Seq ID X00806

ATGGCTTCTATGATATCCTCTTCCGCTGTGACAACAGTCAGCCGTGCCTCTAGGGGGCAA

TCCGCCGCAGTGGCTCCATTCGGCGGCCTCAAATCCATGACTGGATTCCCAGTGAAGAAG

GTCAACACTGACATTACTTCCATTACAAGCAATGGTGGAAGAGTAAAGTGC

*Solanum* chilense (tomatillo) Polygalacturonase Promoter

GTACCATCACATAATTGAGACGAAGGAATAATTAAGATGAACATAGTGTTTAATTAGTAATGGATGGGTAGTAAATTTATTTATAAATTATATCAATAAGTTAAATTATAACAAATATTTGAGCGCCATGTATTTTAAAAAATATTAAATAGTTTGAATTTAAAACCGTTAGATAAATGGTCAATTTTGAACCCAAAAGTGGATGAGAAGGGTATTTTAGAGCCAATAGGGGGATGAGAAGGATATTTTGAAGCCAATATGTGATGGATGAAGGATAATTTTGTATCATTTCTAATACTTTAAAGATATTTTAGGTCATTTTCCCTTCTTTAGTTTATAGACTATAGTGTTAGTTCATCGAATATCATCTATTATTTCCGTCTTAAATTATTTTTTATTTTATAAATTTTTTAAAAATAAATTATTTTTTCCATTTAACTTTGATTGTAATTAATTTTTAAAAATTACCAACATATAAATAAAATTAATATTTAACAAAGAATTGTAACATAATATTTTTTTAATTATTCAAAATAAATATTTTTAAACATCATATAAAAGAAATACGACAAAAAAATTGAGACGGGAGAAGACAAGCCAGACAAAAATGTCCAAGAAACTCTTTCGTCTAAATATCTCTCATCCAAACTAATATAATACCCATTATAATTAACCATATTGACCAACTCAAACCCCTTAAAATCTATAAATAGACAAACCCTTCCCATACCTCTTATCATAAAAAAAATAATAATCTTTTTCAATAGACAAGTTTAAAAACCATACCATATAACAATATATCGCGATCGC
